# Real-Time Measurements of Gas-Phase Medium-Chain Chlorinated Paraffins Reveal Daily Changes in Gas-Particle Partitioning Controlled by Ambient Temperature

**DOI:** 10.1021/acsenvironau.5c00038

**Published:** 2025-06-05

**Authors:** Daniel John Katz, Bri Dobson, Mitchell Alton, Harald Stark, Douglas R. Worsnop, Manjula R. Canagaratna, Eleanor C. Browne

**Affiliations:** † Department of Chemistry and Cooperative Institute for Research in Environmental Sciences, 1877 University of Colorado Boulder, Boulder, Colorado 80309, United States; ‡ 53777 Aerodyne Research, Inc., Billerica, Massachusetts 01821, United States; § Institute for Atmospheric and Earth System Research/Physics, University of Helsinki, Helsinki 00014, Finland

**Keywords:** chlorinated paraffins, polychlorinated alkanes, *in situ* measurements, chemical ionization mass spectrometry, atmospheric chemistry, gas-particle partitioning, organic pollutants

## Abstract

Chlorinated paraffins (CPs) are synthetic polychlorinated *n*-alkanes produced as mixtures of a range of C_*x*_Cl_*y*_H_2*x*–*y*+2_ formulas. CPs have numerous industrial applications but are toxic, long-lived, and environmentally ubiquitous with environmental releases occurring throughout their production, use, and disposal. Short-chain chlorinated paraffins (SCCPs, C_10–13_) have been regulated by the United States Environmental Protection Agency since 2009 and by the Stockholm Convention since 2017. SCCP regulation is expected to cause increased production of medium-chain chlorinated paraffins (MCCPs; C_14–17_), which are currently under consideration for Stockholm Convention regulations. Thus, there is a need to improve the understanding of MCCP environmental transport, distribution, and fate. Existing measurements are limited in their spatial and temporal coverage. Measurements of CP atmospheric loading are particularly scarce. Historically, these measurements have required long sampling times, obscuring the temporal behavior of atmospheric CPs. We report real-time *in situ* measurements of 18 gas-phase MCCPs. These measurements were made in the United States Southern Great Plains with nitrate ion chemical ionization mass spectrometry (NO_3_–CIMS). The estimated average lower-limit concentration of MCCPs is on the order of single-digit ng/m^3^. MCCP diel behavior is partially explained by gas-particle partitioning with implications for MCCP transport and lifetimes.

## Graphical abstract

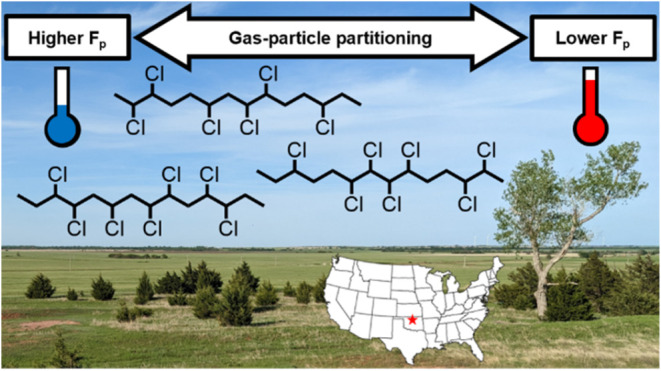

## 1 Introduction

Chlorinated paraffins (CPs) are mixtures of polychlorinated *n*-alkanes and are categorized by their alkyl chain length as short-chain (SCCPs, C_10_–C_13_), medium-chain (MCCPs, C_14_–C_17_), or long-chain (LCCPs, C_≥18_). CPs are synthesized by unselective chlorination of *n*-alkanes, producing a complex mixture containing many isomers of each C_*x*_Cl_*y*_ congener group.^1–3^ Over 1 Mt/year of CPs are produced for use as metalworking fluids, high temperature and pressure lubricants, flame retardants, and plasticizers.^1,2^ An estimated 5.2 Mt of CPs have been released into the environment^4,5^ and CPs are routinely detected in air, water, soils, and biota,^1,6–9^ often at concentrations exceeding other persistent organic pollutants (POPs).^1,10,11^ Due to toxicity to humans and wildlife, environmental persistence, potential for bioaccumulation, and propensity for long-range atmospheric transport^1,5,9,12^ SCCPs were added to Annex A of the Stockholm Convention on POPs in 2017.^1,2,13^ MCCPs remain unregulated under the Stockholm Convention and are expected to increase in production as substitutes for SCCPs.^1,2,13,14^ MCCPs also meet the criteria to be listed under the Stockholm Convention,^5,15^ but understanding of the atmospheric transport and environmental distribution of MCCPs, which is a prerequisite for tighter regulation,^2,14^ has been limited by a severe lack of measurements.^1,7,12,13^

The atmospheric behavior of MCCPs is poorly constrained. Accurate quantification of MCCPs is inhibited by the absence of reliable analytical standards.^14,16^ MCCPs are degraded in the atmosphere by hydroxyl radical (OH) oxidation, but oxidation rates, mechanisms, and product distributions have not been reported.^6,13,17^ Gas-particle partitioning is thought to be a key mechanism of long-range atmospheric transport for low volatility species like MCCPs^13,18,19^ and may increase atmospheric lifetimes by shielding MCCPs that partition onto particles from OH oxidation.^20^ While atmospheric measurements confirm that MCCPs partition into the particle phase,^17–19,21–26^ models have been unable to fully describe the observed gas-particle partitioning of MCCPs.^18,19,26^ One obstacle in modeling MCCPs is that CP mixtures contain many isomers^27^ which can have widely differing physicochemical properties.^2,3,28^ Model predictions of these properties have been reported in several studies,^13,28–30^ but direct measurements are scarce.^3,31^ These studies suggest that MCCPs are semivolatile, but MCCP volatilities vary widely with congener group vapor pressures in the range of 10^0^ to 10^–12^ Pa while vapor pressures for a given molecular formula can differ by multiple orders of magnitude depending on the specific isomer.

Atmospheric measurements of MCCPs have typically been made by collecting samples on either polyurethane foam or glass fiber filters followed by offline analysis with gas chromatography– or liquid chromatography–mass spectrometry (e.g., see refs 13,18,19,23,24,32 ). The highest ambient MCCP concentrations are typically detected near industrial areas with maximum concentrations on the order of 10^5^ pg/m^3^.^13,21,25,38^ MCCP concentrations tend to decrease rapidly with increasing distance from point sources,^1,9,34–36^ but the detection of MCCPs at single-digit pg/m^3^ concentrations in both the Arctic^37^ and Antarctic^18,19^ demonstrates their potential to undergo long-range atmospheric transport. MCCP measurement techniques have low temporal resolution with passive sampling techniques requiring sample collection times of 30–90 days (e.g., see refs 13,23,26,34 ). Sample collection times for active samplers are typically days to months (e.g., see refs 18,19,23–25 ) although several studies report MCCP concentrations with sample collection times on the order of hours.^22,38,39^ These measurements have confirmed the ubiquity of atmospheric MCCPs but are unable to capture MCCP behavior occurring on faster time scales.

More thorough characterization of the distribution and fate of atmospheric MCCPs requires highly temporally resolved measurements that can be used to directly assess MCCP behavior over short time scales (minutes). Expanding the limited spatial coverage of MCCP measurements is also necessary. In this work, we report the first real-time observations of gas-phase MCCPs which were made with a nitrate anion chemical ionization mass spectrometer (NO_3_–CIMS). Detected congener groups contain 14–17 carbon atoms and 5–9 chlorine atoms. These observations were made at the Department of Energy Atmospheric Radiation Measurement Southern Great Plains site in Oklahoma, USA, and represent the first reported detection of atmospheric MCCPs in North America. The sensitivity and high temporal resolution of *in situ* mass spectrometry allow for characterization of the diel behavior of atmospheric MCCPs.

## 2 Experimental Methods

### 2.1 Boundary Layer Gradients in New Particle Formation Campaign

The Boundary Layer Gradients in New Particle Formation campaign^40^ was conducted between 28 April and 31 May 2023 in the Guest Instrumentation Facility of the Atmospheric Radiation Measurement Southern Great Plains site (SGP) in Lamont, Oklahoma, USA (36°36′26.3592″N, 97°29′15.5148″W). Land surrounding the SGP site is used mostly for intensive agriculture of livestock and crops (assessed with USDA-NASS Cropland Data Layer).^41^ In addition, natural gas and petroleum are extracted at several small nearby sites which are located mainly to the west of SGP. The site is rural with only small cities (pop. <50,000) within 100 km. Metropolitan areas including Tulsa, Oklahoma City, and Wichita are all >100 km from the site. However, air masses from larger urban centers at a greater distance from the site, such as Dallas-Fort Worth, occasionally arrive at the site.^42^ A detailed description of the site is provided by Sisterson et al.^43^

### 2.2 NO_3_-CIMS Measurements

Gas-phase molecules were measured with a chemical ionization high-resolution time-of-flight mass spectrometer (CI-HToF-MS, Tofwerk AG and Aerodyne Research Inc.) equipped with an Eisele type NO_3_^–^ inlet (NO_3_–CIMS).^44^ The NO_3_–CIMS has frequently been used to measure highly oxygenated organic molecules and acids due to the tendency of NO_3_^–^ to cluster with polar species.^45,46^ The Eisele inlet is optimized for measuring low volatility compounds with minimal inlet losses and is considered essentially “wall-less.”^45^ A mass flow controller passed clean air from a clean air generator (Model 737–12, Aadco Instruments Inc.) over nitric acid (70% w/w, Sigma-Aldrich) at a flow rate of 10 standard cubic centimeters per minute. The nitric acid-laden clean air was carried into the inlet by a 25 standard liters per minute sheath flow of HEPA-filtered ambient air generated by a blower fan. Ambient air was sampled at a flow rate of 10 standard liters per minute through a 100 cm stainless steel tube with an internal diameter of 11/16”. To prevent insects from entering the inlet the outer end of the inlet tube was covered with two meshes, one stainless steel and one poly­(tetrafluoroethylene). Laboratory experiments confirmed that the addition of the inlet meshes did not increase MCCP signals. An X-ray source (L12536 PhotoIonBar, Hamamatsu Photonics K.K.) was used to generate NO_3_^–^ reagent anions. Ionization of ambient gases occurs in the inlet at atmospheric pressure. All MCCPs were detected as clusters with NO_3_^–^. Ionic clusters entered the mass spectrometer through a 0.3 mm pinhole. The flow rate through the pinhole was approximately 1 liter per minute. The NO_3_–CIMS was operated in “medium mass” mode, which enhances transmission of ions in the range *m*/*z* 50–300. Mass spectra were collected at 1 Hz over the range of *m*/*z* 11–1123 with a resolving power of approximately 5000. The data presented in this work were collected by the NO_3_–CIMS between 6 May and 31 May 2023.

### 2.3 Data Analysis and Quantification

Measurements were postprocessed in IGOR Pro 9.02 (Wavemetrics, Lake Oswego, OR) using Tofware v4.0.0.^47,48^ Data were preaveraged to a 5 min time scale. Peak fitting to MCCPs is shown in Figure S1 with correlations among MCCP isotopologues in Table S1 and discussion of peak fitting and quantification in Section S1. A predeployment mass spectrum showing the lack of MCCPs prior to the field campaign is shown in Figure S2 and discussed in Section S2. All signals were normalized to the sum of the NO_3_^–^, (H_2_O)­NO_3_^–^, (HNO_3_)­NO_3_^–^, and (HNO_3_)_2_NO_3_^–^ reagent ion signals. An instrument transmission function was not applied. This work focuses on MCCPs; measurements of other molecules will be reported in a future publication. A laboratory calibration was performed to quantify the sulfuric acid measured by the NO_3_–CIMS. The details of this calibration have been described previously.^45,46^ Charging of sulfuric acid by NO_3_^–^ occurs near the collision limit^49^ which enables the use of a collision-limited rate coefficient, *k*_ion_, that determines the maximum theoretical sensitivity of the NO_3_–CIMS.^45^ The residence time in the inlet was estimated to be approximately 200 ms. To estimate MCCP concentrations, the measured *k*_ion_ = 2 × 10^–10^ cm^3^/s was used and inlet losses of MCCPs were assumed to be negligible. Direct measurement of the NO_3_–CIMS sensitivity to MCCPs is infeasible because there are few available analytical standards, and the variability of carbon chain length distribution and degree of chlorination between MCCP mixtures means there is no guarantee that the sensitivity to any particular standard will be representative of the sensitivity to the mixture(s) measured in this work.^14,16^ The actual sensitivity of the NO_3_–CIMS to MCCPs is likely lower than the theoretical maximum.^50^ Correcting for the influences of inlet losses, charging efficiency, cluster fragmentation, and/or ion transmission on the MCCP signals would increase the calculated MCCP concentrations, and therefore the MCCP concentrations reported here are lower-limit estimates. Accounting for these effects would require measurements of the isomeric distribution of MCCP mixtures as well as the relevant physicochemical properties of each isomer, neither of which are possible with current techniques.^2,32^ The potential range of instrument sensitivity is discussed further in Section S3. In addition, the NO_3_–CIMS resolving power of approximately 5000 is insufficient to fully resolve MCCP congener groups with overlapping isotope distributions.^16,32,51^ The inability to completely resolve these signals increases the uncertainty in MCCP concentrations and may prevent detection of compounds with especially low signals. However, the most concentrated MCCP congener groups dominate the signals at the unit *m/*z where they appear and can still be reliably quantified (Section S1). Based on the Allan variance of the MCCP signal timeseries, we estimated that averaging on a 5 min time scale results in a limit of detection for individual MCCP congener groups on the order of tens of pg/m^3^, which roughly corresponds to part per quadrillion mixing ratios. We note that, due to the assumptions involved in our calibration, this limit of detection value is a lower limit.

### 2.4 Supporting Analysis

Meteorological measurements including radiation, temperature, wind speed and direction, relative humidity, and boundary layer height are made routinely at the SGP site.^52–54^ An aerosol chemical speciation monitor (Aerodyne Research Inc.) measured the bulk composition of nonrefractory aerosol.^55^ The aerosol chemical speciation monitor organic aerosol mass loading was used for estimates of the organic particle phase associated with gas-particle partitioning of MCCPs. In order to investigate potential sources of MCCPs at SGP, we used nonparametric wind regression (NWR) analysis, which is described in detail by Henry et al.^56^ Briefly, NWR uses the measured concentration of a pollutant and the corresponding wind speed and wind direction to assess if certain wind sectors are associated with higher pollutant concentrations. This is accomplished by using smoothing kernels to spread the observed concentrations over a fine grid of wind speed and wind direction. This allows pollutant concentrations to be estimated for combinations of wind speed and wind direction which were not actually observed based on nearby combinations of wind speed and wind direction which were observed. The sum of the concentrations of the six most abundant observed MCCPs was used as the concentration for the NWR analysis because the sum concentration is more representative of total MCCPs and provides higher signal-to-noise than the concentration of a single MCCP congener group. NWR plots were made with Zefir v3.7,^57^ a wind and trajectory analysis tool based in Igor Pro.

## 3 Results and Discussion

### 3.1 MCCP Congener Group Distribution and Temporal Trends

This work represents the first demonstration of NO_3_^–^ chemical ionization to detect MCCPs. However, it is not unexpected that the technique is sensitive to MCCPs because similar ionization schemes, particularly Cl^–^ and Br^–^ atmospheric pressure chemical ionization, have been successfully employed for offline quantification of MCCPs in various environmental samples.^32,33,58^ Both halide and nitrate ionization schemes are sensitive to highly polarizable compounds such as MCCPs. As with formation of Cl^–^ and Br^–^ adducts in atmospheric pressure chemical ionization, the formation of NO_3_^–^ adducts is a soft ionization scheme which transfers little energy to analytes. This means that NO_3_^–^ chemical ionization likely avoids significant fragmentation of MCCPs. Fragmentation has historically been a challenge for accurate quantification of MCCP congener groups.^51^ The NO_3_–CIMS exclusively detects gaseous compounds, and therefore all mentions of MCCP concentrations refer only to the gas phase unless otherwise noted.

Our NO_3_–CIMS measurements allow us to characterize the temporal trends of MCCP congener groups in the gas phase at the SGP site. Detected MCCP congener groups have 14–17 carbon atoms and 5–9 chlorine atoms. Estimated average concentrations of MCCP congener groups during the deployment at SGP are presented in Figure 1. Average, lower quartile, median, upper quartile, and maximum concentrations for the six most abundant congener groups are presented in Table S2. C_14_ MCCPs have the highest average concentrations. C_15_ MCCPs are present at similar albeit lower concentrations, while C_16_ and C_17_ MCCPs are significantly less abundant. With respect to chlorine atoms, Cl_6_ and Cl_7_ MCCPs are present at the highest average concentrations and detected Cl_9_ MCCPs are present at concentrations which are only slightly higher than the detection limit. The six most abundant congener groups are C_14_Cl_6_H_24_, C_14_Cl_7_H_23_, C_14_Cl_8_H_22_, C_15_Cl_6_H_26_, C_15_Cl_7_H_25_, and C_16_Cl_6_H_28_ with respective average concentrations of 490, 500, 190, 380, 320, and 190 pg/m^3^.

**Figure 1:**
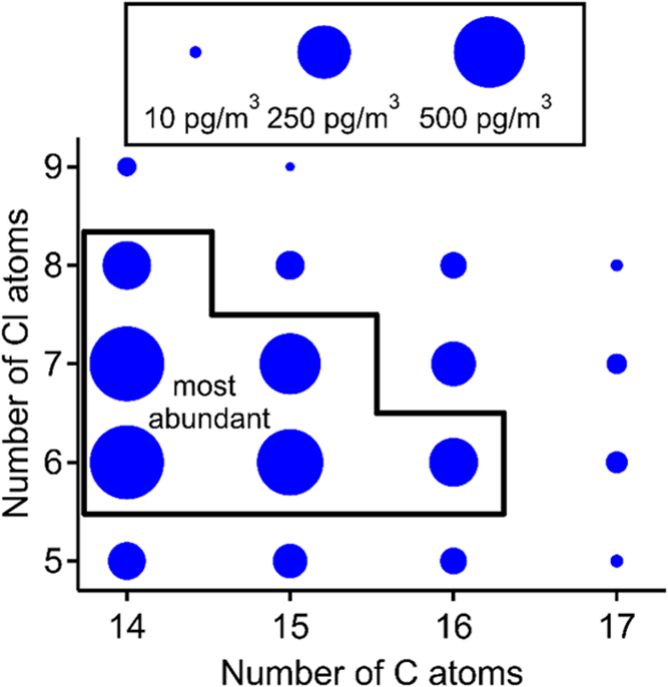
Average mass concentration of each MCCP congener group across the campaign. Marker area is proportional to mass concentration. Congener groups with no corresponding marker were not detected.

Across the campaign, the median and average mass concentrations of total observed MCCPs are respectively 2500 and 3100 pg/m^3^. These concentrations are multiple orders of magnitude higher than the unit pg/m^3^ concentrations measured in remote regions,^18,19^ suggesting that MCCP concentrations at the SGP site are not solely the result of long-range atmospheric transport. The average MCCP concentration at the SGP site is more comparable to MCCP concentrations measured at a semirural field site in the U.K. (3040 pg/m^3^),^38^ several suburban sites near Dar-es Salaam, Tanzania (5000 pg/m^3^),^34^ and some urban sites in China (Beijing,^59^ 3600 pg/m^3^; Guangzhou,^60^ 3530 pg/m^3^; Dalian City,^61^ 4890 pg/m^3^). It is lower than the average MCCP loadings in nine cities in the Pearl River Delta, China (15,700 pg/m^3^)^22^ and ten cities across mainland China (15,600 pg/m^3^).^21^ Several studies demonstrate that atmospheric MCCP concentrations may differ by orders of magnitude among measurement sites within tens of km of each other,^22,34,35,38,39^ which is consistent with most MCCPs emitted to the atmosphere being transported short distances (∼1–10 km), resulting in environmental MCCP concentrations decreasing rapidly with increasing distance from emission sources.^9,34–36^ Our measurements therefore suggest that there are sources of atmospheric MCCPs nearby the SGP site, but with only a single measurement site it remains unknown how the SGP MCCP concentration compares to other sites in the region or to the regional average. It should be noted that it is difficult to directly compare MCCP concentrations between studies because of the large uncertainties in MCCP measurements and because individual studies may report gas-phase MCCPs, particle-phase MCCPs, or total atmospheric MCCPs depending on which sampling technique(s) were employed.

The trends in congener abundance imply that more volatile congener groups are generally more abundant in the gas phase than less volatile congener groups. A notable exception is Cl_5_ congener groups which are more volatile than Cl_6_ or Cl_7_ groups but are present at lower concentrations. This may be in part because typical MCCP products have high degrees of chlorination such that Cl_≥6_ congener groups are more highly concentrated in MCCP mixtures than Cl_≤5_ congener groups.^2,16^ It is also possible that Cl_≤5_ congener groups are detected at lower concentrations because the ionization efficiency of NO_3_^–^ decreases for less chlorinated congener groups, which is a bias that is known to affect electron capture negative ionization mass spectrometry measurements of MCCPs.^32^ The most abundant congener groups at SGP are C_14–15_ and Cl_6–7_, which is similar to other sites. Previously reported gas-phase MCCP distributions consistently show that, with respect to carbon atoms, C_14_ and C_15_ congener groups are more abundant than longer-chain MCCPs.^18,19,23–26^ There is more variability in gas-phase chlorine atom congener groups with some studies also finding that Cl_6_ and Cl_7_ congener groups dominate^18,25^ and some finding that congener groups with fewer chlorine atoms are more abundant.^19,23,24,26^

The high temporal resolution of the NO_3_–CIMS allows us to investigate real-time MCCP diel behavior in the atmosphere. Figure 2 shows the median diel profiles for the six MCCP congener groups with the highest average mass concentrations. These congener groups account for approximately 66% of the total observed MCCP mass concentration. Gas-phase concentrations of all MCCP congener groups are higher during the day and decrease at night. The relative and absolute day/night differences vary substantially among different congener groups. For example, C_14_Cl_7_H_23_ has the highest median nighttime concentrations, but in the middle of the day C_14_Cl_6_H_24_ peaks at a higher median concentration due to a substantially larger daytime concentration increase. The variations in gas-phase MCCP concentrations between daytime and nighttime have not been previously reported.

**Figure 2:**
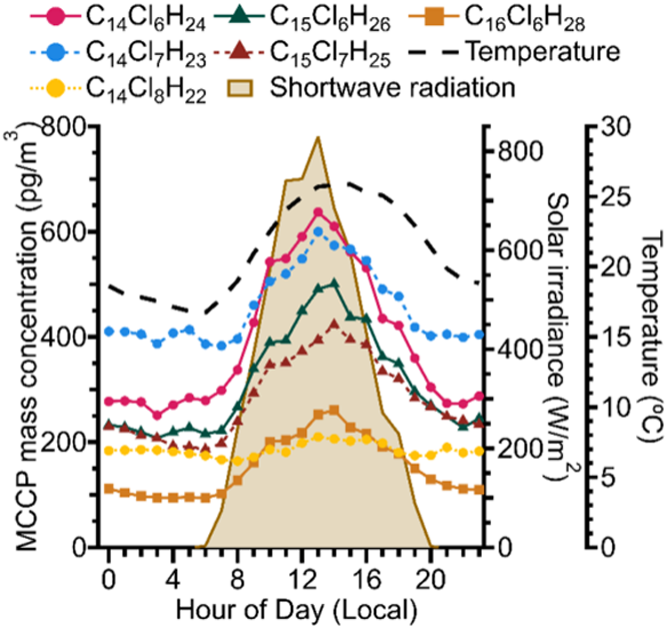
Diel profiles showing the median hourly mass concentration of the six most abundant MCCPs (traces with symbols), median hourly surface temperature (dashed trace), and median hourly shortwave solar irradiance (shaded area) throughout the campaign. The variation in shortwave solar irradiance over a typical day gives an indication of when photochemistry is active at the site. MCCPs with the same number of carbon atoms have the same markers, MCCPs with the same number of chlorine atoms have the same line style.

Timeseries of the six most abundant MCCPs over several consecutive days are shown in Figure 3. Timeseries of the concentrations of the six most abundant MCCPs and the sum concentration of all MCCPs over the entire campaign can be found in Figures S3 and S4. The MCCP timeseries show temporal evolution in both concentration and distribution of MCCPs. The timeseries are broadly similar, with all congener group concentrations having similar temporal behavior and peaking on the same day. The daytime peak in gas-phase MCCP concentrations shown in the diel profiles is apparent in the timeseries. However, the timeseries also demonstrate that the magnitudes of the peak concentrations change over time. During the campaign the highest MCCP concentrations were measured on May 6, coinciding with the highest observed surface temperatures. Following May 6, the peak concentrations decrease on each subsequent day until they increase on May 12. It is also evident from the timeseries that the distribution of MCCP congener groups changes over time. For example, the peak concentrations of the C_14_Cl_6_H_24_, C_15_Cl_6_H_26_, and C_14_Cl_7_H_23_ congener groups differ widely on May 6 with the concentration of C_14_Cl_6_H_24_ nearly a factor of 2 higher than the concentration of C_14_Cl_7_H_23_. Over the next 5 days the differences in daily peak concentrations diminish and on May 11 all three congener groups have similar concentrations. The concentrations diverge again on May 12, coinciding with the increase in peak concentration. The pattern in which trends are consistent over several days and then change substantially repeats several times during the campaign and may be associated with influences from different air masses or sources of MCCPs (discussed further in Section 3.3).

**Figure 3:**
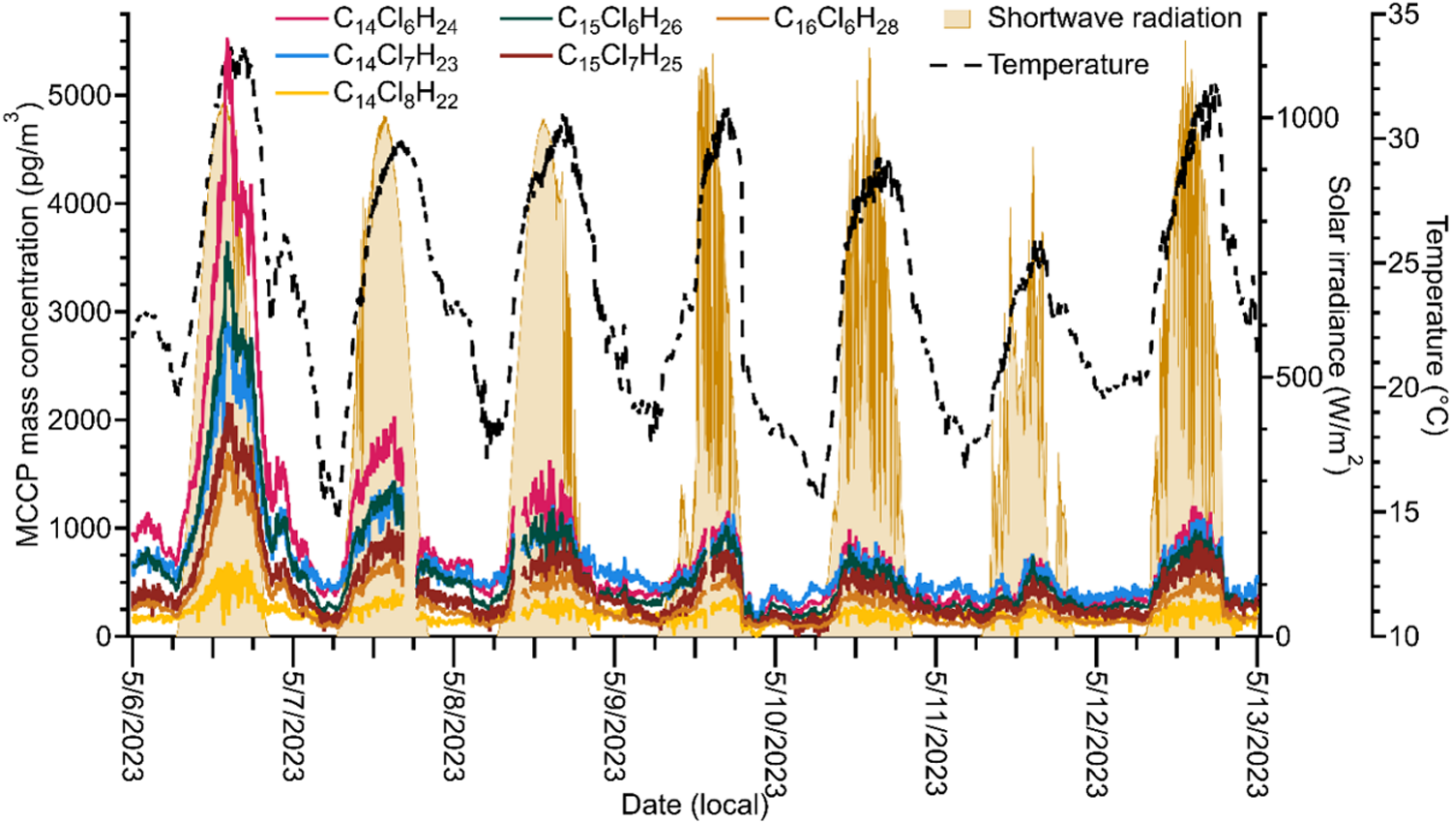
Timeseries of the six MCCPs with highest average mass concentration (solid traces), surface temperature (dashed trace), and shortwave solar irradiance (shading).

The morning increase in MCCP concentrations implies there is a gas-phase MCCP source during the day. If the total gas-phase MCCP loading in the boundary layer were constant, then morning increases in boundary layer height should dilute MCCPs and decrease daytime concentrations. The median diel profile of boundary layer height is shown in Figure S5. Further, the oxidation of MCCPs by OH should also decrease daytime MCCP concentrations. There are no known chemical pathways which form MCCPs in the ambient atmosphere. Similarly, the nighttime decreases in MCCP concentrations are inconsistent with oxidation chemistry because OH oxidation only occurs to any appreciable extent during the day while nighttime oxidants NO_3_ and O_3_ are presumably unreactive with MCCPs. These nighttime decreases therefore suggest that there is a process which removes MCCPs from the gas phase at night. The daytime increase and nighttime loss of MCCPs is most likely a result of MCCPs partitioning between condensed phases and the gas phase in accordance with daily temperature changes.

### 3.2 MCCP Gas-Particle Partitioning

Gas-particle partitioning is a key atmospheric process which controls the long-range atmospheric transport and fate of CPs as well as routes of human exposure.^13,18,19,21,61^ The Clausius–Clapeyron equation predicts a linear relationship between the natural log of vapor pressure and inverse temperature for a compound partitioning between the gas phase and a condensed phase. In order to assess whether the observed behavior of MCCPs was consistent with temperature-controlled partitioning, the natural log of congener group partial pressure in pascals, ln *P*_MCCP_, of each of the six most abundant congener groups was plotted versus inverse temperature, 1/*T*, for measurements made on May 6–9, as shown in Figure 4. This period of several days was chosen for this plot because it is short enough that it can be assumed that total MCCP loadings are fairly constant, but there is still a relatively wide range of observed temperatures (∼14–34 °C). Over a longer time period changes in total MCCP loading would cause spread in the plot even if the gas-phase MCCP concentrations are controlled by partitioning in the short term. Figure 4 confirms that there is a linear relationship between the ln *P*_MCCP_ and 1/*T* for each congener group, suggesting that temperature-dependent partitioning controlled MCCP concentrations during this period. During May 6–9 the average organic aerosol loading measured by the aerosol chemical speciation monitor was 4.3 ± 2.1 μg/m^3^ (range of 0.7–8.6 μg/m^3^) with higher organic aerosol loading tending to correspond to ln *P*_MCCP_ values which fall below the line of best fit and lower organic aerosol loading tending to correspond to ln *P*_MCCP_ values which fall above the line of best fit. This is consistent with increased particle loading causing a decrease in gas-phase MCCPs while total atmospheric MCCPs remain constant. The role of organic aerosol loading in controlling MCCP concentrations is discussed further below. Other periods in the campaign show similar linear trends which are shifted along the *y*-axis in response to changes in total MCCP loading. The correlation is particularly strong for the C_15_Cl_6_H_26_, C_15_Cl_7_H_25_, and C_16_Cl_6_H_28_ congener groups, as demonstrated by the higher *R* ^2^ values (Figure 4d–f). The correlation is weaker for the C_14_Cl_8_H_22_ congener group, which may be due to its lower abundance and signal-to-noise ratio compared to the other congener groups in Figure 4, but there is nonetheless a clear linear relationship (Figure 4c).

**Figure 4:**
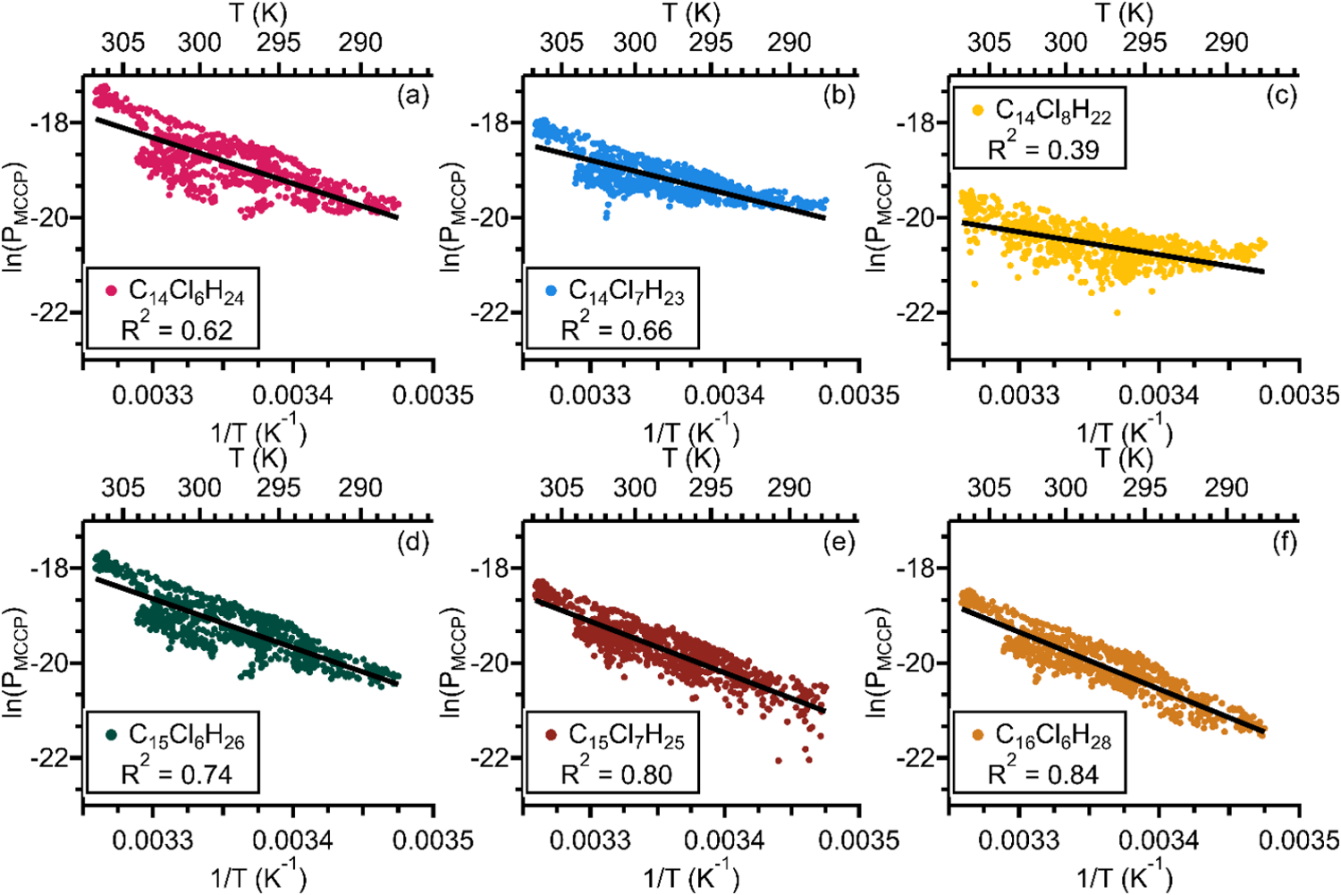
Plot of natural log of MCCP partial pressure in pascals versus inverse temperature (bottom axis, K^–1^) and temperature (top axis, K) for 6–9 May (*n* = 823 measurements). Black lines show the line of best fit. The high *R* ^2^ values show that there is a linear relationship between ln *P*_MCCP_ and 1/*T*.

According to the Clausius–Clapeyron equation, the slope of the linear relationship between the natural log of congener group concentration and inverse temperature is related to Δ*H*_vap_ (kJ/mol) by

1 $$\mathrm{slope}=\frac{-\Delta H_{\mathrm{vap}}}{R}$$

where *R* is the ideal gas constant (8.314 × 10^–3^ kJ/mol·K). Thus, the slopes of the linear regressions shown in Figure 4 provide estimations of the congener group effective Δ*H*_vap_. We refer to this quantity as effective Δ*H*_vap_ because (1) this calculation requires making assumptions which are not true of the ambient atmosphere, in particular that total MCCP loading is constant and that gas-phase MCCP concentrations are affected only by gas-particle partitioning, and (2) the MCCP concentrations we present are lower-limit concentrations with substantial uncertainty (Section 2.3). Additionally, this analysis assumes that equilibrium between the gas- and particle-phase concentrations has been reached, which may not be true on short (minute long) time scales due to mass transfer limitations or if total MCCP loadings are too low for saturation of the gas phase. Given these uncertainties, we do not interpret these values quantitatively. Instead, we focus on the general magnitude of the slopes compared to estimated and measured values of Δ*H*_vap_ for the six congener groups shown in Figure 4. For C_14_Cl_6_H_24_, C_15_Cl_6_H_26_, C_15_Cl_7_H_25_, and C_16_Cl_6_H_28_, the effective Δ*H*_vap_ calculated from the best fit line lies between ∼80 and ∼100 kJ/mol. Hammer et al.^31^ measured similar values of 84 kJ/mol for C_14_Cl_6_H_24_ and 94 kJ/mol for C_14_Cl_7_H_23_ but did not report measurements of the other four congener groups. Endo^30^ estimated values for all six congener groups which tend to be slightly higher at around ∼110–125 kJ/mol. For C_14_Cl_7_H_23_ and C_14_Cl_8_H_22_, the respective effective Δ*H*_vap_ of ∼60 and ∼40 kJ/mol from the regression analysis are lower than expected, which may be related to a breakdown in the assumptions of constant total loading and/or uncertainties in quantification.

The relationship predicted by the Clausius–Clapeyron equations holds for a compound partitioning between the gas phase and any condensed phase, and it is likely that MCCPs partition between the gas phase and multiple condensed phases including soils and particles. However, it is well-established that atmospheric MCCPs partition into the particle phase,^18,19,23–26^ and several aspects of the diel variation in MCCP concentration are consistent only with gas-particle partitioning. The temporal resolution of our measurements provides evidence to support that gas-particle partitioning controls MCCP concentrations at this site. Figure 4 shows that MCCP concentrations respond to changes in surface temperature on a 5 min time scale. This time scale is faster than soil or water temperatures change in response to surface temperature, meaning partitioning from these reservoirs alone cannot explain the trends in MCCP concentrations. Similarly, typical gas-phase deposition time scales are too long to explain the rapid afternoon decrease in MCCP concentrations, suggesting that partitioning to particles is the dominant removal process.

Several models have been developed to explain gas-particle partitioning of MCCPs and other SVOCs.^62–64^ Studies comparing model predictions and direct measurements of MCCP congener group particle fractions, *F*_p_, have shown that current models partially explain MCCP gas-particle partitioning but are unable to fully capture partitioning of all MCCP congener groups across a range of ambient conditions.^19,24,26^ Without measurements of particle-phase MCCPs, we cannot directly calculate *F*_p_, but if gas-particle partitioning is controlling MCCP concentrations at the SGP site, we would expect to observe a trend in which we measure higher MCCP concentrations when model-predicted *F*_p_ are lower. We used the physicochemical properties listed in Table S3 to calculate *F*_p_ based on the Pankow model^62,65^ (Section S7). The organic particle phase available for partitioning was determined from measurements of organic aerosol loading made with an aerosol chemical speciation monitor. Figure S6 shows measured MCCP concentrations plotted against calculated *F*_p_ for the entire campaign. Higher MCCP concentrations are associated with lower *F*_p_, which suggests that gas-particle partitioning has a significant role in controlling MCCP concentrations at SGP. While it is likely that air–water and air–soil partitioning affect the total atmospheric flux of MCCPs (see Section 3.3), the time scale of changes in gas-phase MCCP concentrations and the agreement with trends predicted by a gas-particle partitioning model suggest that the variability in MCCP concentrations we observe is due primarily to gas-particle partitioning.

Partitioning of MCCPs between the gas phase and condensed phases likely explains both the diel profile and the longer-term temporal behavior of atmospheric MCCPs. The morning increase in MCCP concentrations is a result of the increase in temperature which causes a larger fraction of MCCPs to partition into the gas phase. Changes in MCCP gas-particle partitioning controlled largely by temperature have been previously observed on a seasonal scale by Ma et al.,^25^ who found that MCCP *F*_p_ decreased substantially from spring to summer principally due to increased summer temperatures. Partitioning behavior also explains the different magnitude of daytime increases among congener groups. According to the Clausius–Clapeyron equation, when temperature increases the lower molecular weight congener groups with smaller Δ*H*_vap_ will have a larger increase in *P*_vap_ than higher molecular weight congener groups. This causes more volatile congener groups to have a larger increase in daytime concentration compared to nighttime concentration.

Assuming that measurements over the period shown in Figure 3 reflect one source of MCCPs which is not being replenished, the changes in MCCP distribution over time may be explained by the different partitioning behavior of each congener group. The peak concentrations of higher volatility congener groups included in Figures 3 and S3 decrease by a larger fraction each day than the peak concentrations of the lower volatility congener groups. This may be because higher fractions of more volatile MCCPs are present in the gas phase where they can be lost to OH oxidation and/or gas-phase deposition. For the less volatile MCCPs only a small fraction is present in the gas phase, and therefore their concentrations are not affected as quickly by OH oxidation or deposition. Although we do not have measurements of particle-phase MCCPs at SGP, measurements at other sites suggest that congener groups with more carbon and/or chlorine atoms tend to have lower volatilities and have higher affinity for the particle phase,^18,19,23,25^ and it is likely that lower volatility species are also more abundant in the particle phase at SGP. MCCP transport models suggest that particle-phase degradation is a non-negligible loss process of MCCPs, but it occurs at much slower rates than gas-phase OH oxidation.^5^ While slower particle-phase degradation would increase the atmospheric lifetime of all MCCPs that partition onto particles, the effect would be greater for less volatile MCCPs with higher *F*_p_. Beyond changing the distribution of MCCP congener groups, this partitioning behavior could alter the composition of isomers within a given congener group over time as more volatile isomers spend more time in the gas phase and are lost more quickly to OH oxidation and deposition. If isomers are lost at different rates depending on their volatility then the partitioning properties of MCCP congener groups could depend on atmospheric aging, which may have implications for comparisons of MCCPs measured nearby MCCP sources with those measured in remote regions where MCCP mixtures may have undergone significant aging. While the high temporal resolution of the NO_3_–CIMS allows us to identify the changes in ratios of gas-phase MCCPs, fully testing this hypothesis would require measurements of particle-phase MCCPs.

### 3.3 Possible Sources of MCCPs

As discussed in Section 3.1, the relatively high concentration of atmospheric MCCPs at the SGP site implies that there are sources of MCCPs in the region. With measurements only at a single site, we cannot characterize MCCP spatial distributions in the region, which makes it difficult to identify potential sources of the observed atmospheric MCCPs. However, there are some occasions where the observed MCCP concentrations are inconsistent with the trend predicted by partitioning alone, hinting at possible sources. A clear example occurs from 18:00–20:00 LT on May 13 (Figure S7) when a change in wind direction coincides with a substantial decrease in temperature while the organic aerosol loading remains fairly constant. Based on partitioning alone we would expect a decrease in temperature to lead to an increase in partitioning to condensed phases and a corresponding decrease in gas-phase MCCP concentrations, but the temperature decrease is accompanied by an increase in MCCP concentrations. This observation is consistent with an air mass with higher total MCCP loadings arriving at the site and therefore provides some evidence that MCCPs are transported to site rather than emitted at the site or very nearby.

The NWR plot of the six most abundant MCCPs is shown in Figure 5. For comparison, a wind rose and a pollution rose are presented in the Supporting Information (SI) (Figures S8 and S9). The NWR plot demonstrates that significant MCCP concentrations are observed for nearly every combination of wind speed and direction. This is consistent with distributed sources around the site. There are certain combinations of wind speed and direction, particularly high winds from the southwest, which are associated with higher concentrations of MCCPs. Due to the limited time period of this campaign (approximately 1 month), this finding does not necessarily imply that there is a greater source of MCCPs which corresponds to those wind sectors. The highest MCCP concentrations were observed on May 6, which was also the day with the highest surface temperatures and the only day with high winds from the southwest. Based on the partitioning behavior of MCCPs we would expect to measure higher gas-phase MCCP concentrations from any given direction on a day with higher temperatures. Because there are no observations of this wind sector with lower surface temperatures, we cannot conclusively say whether the high measured concentrations result from increased temperatures, the combination of wind speed and wind direction, or both. However, as discussed in Section 3.2, the linear relationship between the natural log of MCCP concentrations and inverse temperature observed on May 6 and the following days (Figure 4) suggests that the decrease in MCCP concentrations after May 6 is consistent with the decrease in temperature even if total MCCP loading was approximately constant.

**Figure 5:**
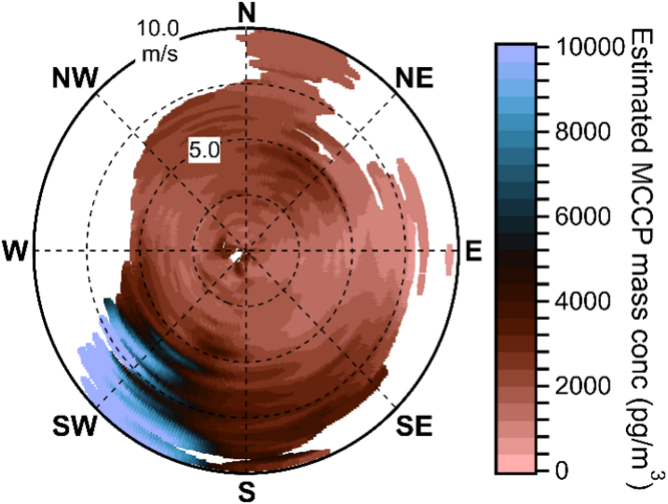
Nonparametric wind regression analysis of the sum of concentrations of the six most abundant MCCP congener groups across the entire campaign. Certain combinations of wind speed and direction, particularly high winds from the southwest, are associated with higher concentrations of MCCPs, but MCCPs are measured with every observed combination of wind speed and direction. White areas of the plot represent combinations of wind speed and wind direction for which there was insufficient information to estimate a corresponding concentration.

MCCPs can be released during their production, use, and disposal,^1^ and due to historical emissions likely already exist as environmental contaminants in this region.^4^ The few environmental measurements of MCCPs in North America have generally focused on aquatic ecosystems.^66–68^ These studies did not investigate atmospheric MCCPs and were carried out at sites >1000 km from the SGP site, so the information they provide about environmental MCCPs is not directly relevant to our MCCP measurements at SGP. Without detailed knowledge of MCCP atmospheric behavior and lifetimes it is difficult to identify potential sources in the region. However, the NWR analysis shows that MCCPs are consistently detected regardless of wind speed and direction. Based on this observation we hypothesize that we are seeing MCCPs from several sources surrounding the SGP site. We speculate that one possibility is that MCCPs are present in wastewater and are introduced to agricultural soils via sewage sludges used as biosolid fertilizers and/or wastewater irrigation.^4^ Li et al.^69^ showed that nearly 70% of MCCP mass loading remained in sewage sludge following wastewater treatment, and MCCPs have been reported in sewage sludges in China,^69^ Australia,^70^ and the United Kingdom.^71,72^ Zeng et al.^73^ connected wastewater irrigation to increased levels of SCCPs in agricultural soils. Biosolid fertilization and wastewater irrigation are both used for agriculture in Oklahoma.^74,75^ MCCPs could be introduced directly into the atmosphere when sewage sludges are spread across soils or when fields are sprayed with wastewater, and following these processes MCCPs present in the top layer of soil could partition into the atmosphere. Once they enter the atmosphere, MCCPs can partition between the gas and particle phases. Biosolid fertilizers are sometimes applied in late spring/early summer before planting summer crops,^76^ which would coincide with the season in which our measurements were made. If there were a nearby application of biosolid fertilizer containing MCCPs on or before May 6 that might explain the higher MCCP concentrations observed on May 6 and the following days. Regardless of the source of the MCCPs we observe, our measurements provide confirmation that atmospheric MCCPs are present in the agricultural Southern Great Plains and warrant further characterization.

### 3.4 MCCP Oxidation Products

We identify numerous MCCPs containing oxygen atoms in their formulas (oxidized MCCPs, oMCCPs), which are most likely products of atmospheric oxidation of MCCPs. These oMCCPs contribute approximately 20% of the average mass concentration of the total observed MCCPs. The most abundant oMCCPs that we detect have C_*x*_Cl_*y*_H_2*x*–*y*+2_O_2_ formulas. Based on analogy to the oxidation mechanisms of *n*-alkanes, we expect that these are most likely hydroperoxides. MCCP hydroperoxide oxidation products could be formed by oxidation initiated by OH H atom abstraction and terminated by reaction of HO_2_ with MCCP-derived peroxy radicals (Figure S10). We also detect oMCCPs which contain N atoms in their formulas. The detection of N-containing oMCCPs implies that another possible fate of MCCP RO_2_ at SGP is reaction with NO to form oMCCPs with organonitrate functional groups (Figure S10). The diel behavior of most oMCCPs (Figure S11) is roughly similar to the unfunctionalized MCCPs, which suggests that they may undergo similar partitioning as unfunctionalized MCCPs. The instrumental resolution is insufficient to fully resolve and identify all oMCCPs which may contribute to the mass spectra. For example, MCCPs which fragment during oxidation could form products of many different chain lengths such that no individual product is concentrated enough to be identified. In addition, some oxidation mechanisms, particularly autoxidation, rapidly form very low volatility products that would likely partition primarily into the condensed phases where they would not be detectable by the NO_3_–CIMS. Thus, the oMCCPs which we observe allow us to hypothesize that some oxidation pathways may be occurring at SGP, but we do not rule out the possibility that MCCPs are also oxidized by different mechanisms that produce oMCCPs which were not detected.

## 4 Atmospheric Implications

MCCPs have been manufactured in the USA for nearly a century^4^ and continue to be used for a wide variety of applications. While the majority of MCCPs are currently produced in China, where most measurements of atmospheric MCCPs have been made, Chen et al.^4^ estimate greater total emissions of MCCPs to the environment in North America than in China largely due to differing applications. Compared to other regions, a higher portion of MCCPs are used in metalworking fluids in North America, and this tends to result in substantial emissions, mostly to wastewater, during use.^4^ Given the historical context and the continued production of MCCPs in the USA^4,15^ it is not unexpected that significant quantities of MCCPs are present in the ambient air. Our measurements confirm that atmospheric MCCPs are present in North America, and the concentrations measured at the SGP site suggest that there are sources of MCCPs in the region. More measurements are required to determine MCCP spatial and temporal distribution throughout regions representing a variety of land use categories and across different seasons. This work provides new insight into atmospheric MCCP behavior with direct observations of temperature-controlled partitioning and the identification of several likely oMCCPs. While oxidation, deposition, and partitioning affect the environmental transport of MCCPs, these processes are poorly constrained due to insufficient fundamental measurements. Our observations of variations in relative congener group abundance suggest that measurements directly probing gas-particle partitioning across a range of aerosol types under highly controlled conditions are required to better understand transport and fate of MCCPs in the environment.

## Supplementary Material



## Data Availability

The data underlying this study are openly available at the Department of Energy (DOE) Office of Science Atmospheric Radiation Measurement (ARM) User Facility Data Center at: https://adc.arm.gov/. Concentration timeseries of select MCCPs can be found at: 10.5439/2562637. Individual data products can be accessed at the DOI provided in the references.

## Notes

Discussion of peak fitting and instrumental sensitivity, mass spectra, concentration estimates, additional timeseries and diel profiles, particle fraction estimates, rose plots, and proposed reaction mechanism (PDF)
