# Supplementary material for: Real-Time Measurements of Gas-Phase Medium-Chain Chlorinated Paraffins Reveal Daily Changes in Gas-Particle Partitioning Controlled by Ambient Temperature
Source: ACS Environ Au. 2025 Jun 5;5(4):415–26. doi: 10.1021/acsenvironau.5c00038 (PMC12272276; doi:10.1021/acsenvironau.5c00038)
Supplement: Supplementary file 1 [file vg5c00038_si_001.pdf]

Supporting Information: Real-time measurements of gas-phase medium chain chlorinated paraffins reveal daily changes in gas-particle partitioning controlled by ambient temperature

Daniel John Katz,[1] Bri Dobson,[1] Mitchell Alton,[2] Harald Stark,[1,2] Douglas R. Worsnop,[2,3] Manjula R. Canagaratna,[2] and Eleanor C. Browne[*1]

[1]Department of Chemistry and Cooperative Institute for Research in Environmental Sciences, University of Colorado Boulder, Boulder, Colorado, 80309 USA

[2]Aerodyne Research, Inc., Billerica, Massachusetts, 01821 USA

[3]Institute for Atmospheric and Earth System Research/Physics, University of Helsinki, Helsinki, Finland

*Corresponding author. Email: eleanor.browne@colorado.edu

S1 – MCCP peak fitting and isotopologue allocation

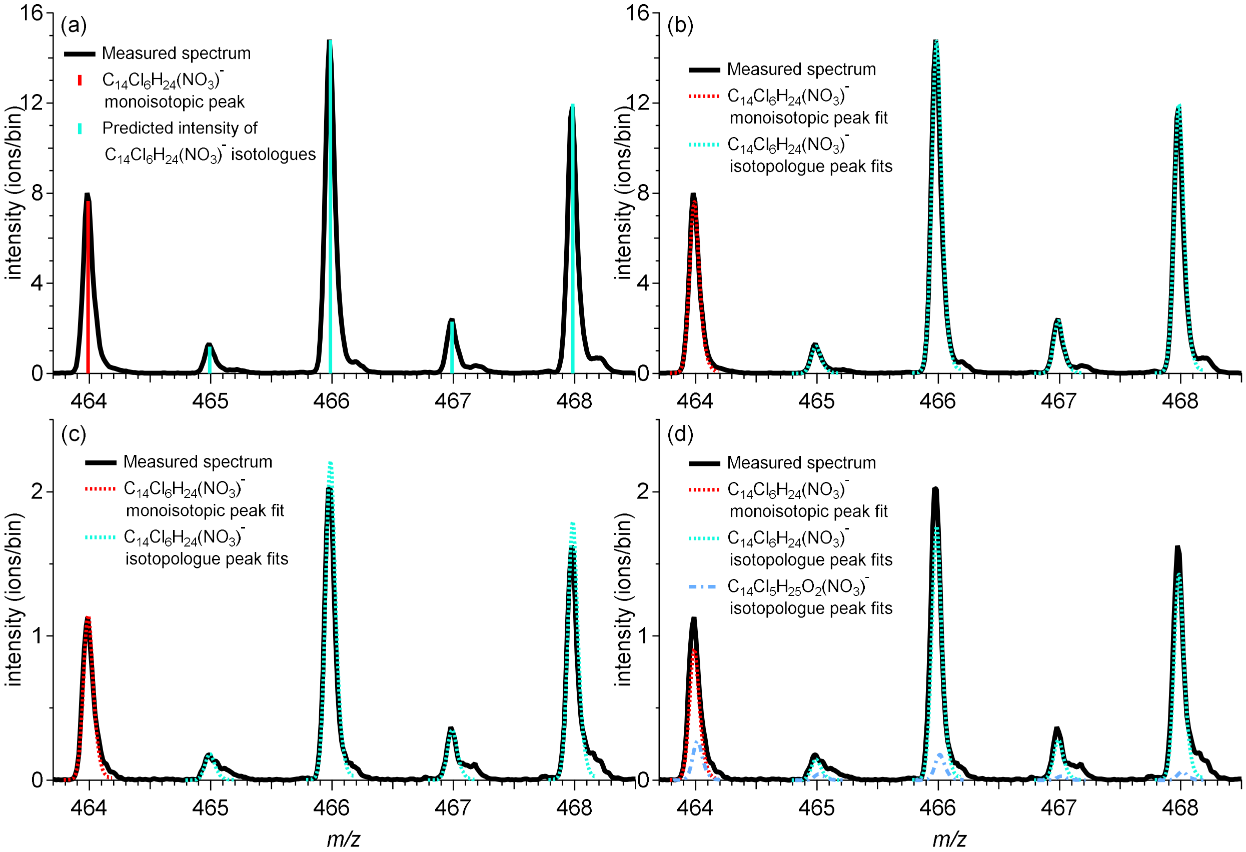

Figure S1: Five-minute average mass spectra (black traces) demonstrating the assignment of peaks to MCCPs and oxidized MCCPs. Panels (a) and (b) show the same spectrum in which $C_{14}Cl_6H_{24}(NO_3^-)$ dominates the MCCP signal. Panel (a) shows the location of the $C_{14}Cl_6H_{24}(NO_3^-)$ monoisotopic ion (red stick) as well as the locations and predicted intensities of isotopologue ions (teal sticks). Panel (b) shows the isotopologue peak fits (teal dashed traces) when $C_{14}Cl_6H_{24}(NO_3^-)$ is fit at *m/z* 464 (red dashed trace). A different spectrum is shown in panels (c) and (d). Panel (c) shows that if $C_{14}Cl_6H_{24}(NO_3^-)$ is assigned at *m/z* 464 (red dashed trace) the fitted isotopic peaks (teal dashed traces) are larger than the observed signal. Panel (d) shows that if an oxidized MCCP, $C_{14}Cl_6H_{24}O_2(NO_3^-)$, is assigned at *m/z* 462 (not shown) then some signal is allocated to its isotopologue ions (blue segmented traces) reducing the signal allocated to the $C_{14}Cl_6H_{24}(NO_3^-)$ monoisotopic ion (red dashed trace). This decreases the size of $C_{14}Cl_6H_{24}(NO_3^-)$ isotopologues fit at higher *m/z* (teal dashed traces), and the fits are no longer greater than the observed signal.

The spectra shown in Fig. S1 demonstrate the assignment and fitting of MCCP peaks using $C_{14}Cl_6H_{24}(NO_3^-)$ as an example. Briefly, we perform constrained peak fitting to allocate signal between overlapping ions. Following established protocols, we determine the isolated peak shape, baseline signal, and the peak width (as a function of *m/z*).[1,2] As shown in Fig. S1a, the exact *m/z* of $C_{14}Cl_6H_{24}(NO_3^-)$ (red stick) agrees well with the location of the measured signal. We fit the monoisotopic ion (Fig. S1b red trace) and the intensities of the isotopologues are calculated based on the stable isotope abundances (Fig. S1b teal traces). Figure S1 shows only the largest $C_{14}Cl_6H_{24}(NO_3^-)$ isotopologues at lower *m/z* (*m/z* 464-468) to enhance readability; all isotopologues with an intensity > 0.1% of the monoisotopic peak are fit.

It is possible to evaluate the contribution of isobaric ions by inspecting the agreement between the theoretically predicted isotopologues and the measured signal. In the spectrum shown in Figs. S1a and S1b $C_{14}Cl_6H_{24}(NO_3^-)$ alone explains the observed MCCP signal. In both (a) and (b) the predicted/fitted intensities at each *m/z* closely match the observed signal, and the agreement is within 1% for the isotopes at *m/*z 466 and 468 which have the highest signal-to-noise and contribute the majority of the $C_{14}Cl_6H_{24}(NO_3^-)$ isotope signal. This is not the case for the spectrum shown in Figs. S1c and S1d. Figure S1c shows the predicted intensity at higher *m/z* if $C_{14}Cl_6H_{24}(NO_3^-)$ is assigned to the negative mass defect peak at *m/z* 464 (red dashed trace), but unlike panel (a) the fitted MCCP signals at higher *m/z* (teal dashed traces) are too large and the fitted signals are approximately 10% greater than the observed signals at *m/z* 466 and 468. This is due to an oxidized MCCP compound which also contributes to the negative mass defect MCCP signal in the spectrum. Panel (d) shows that when $C_{14}Cl_5H_{25}O_2(NO_3^-)$ is fit to the MCCP peak at *m/z* 462 (not shown) then some of the signal at *m/z* 464 is allocated to one of its isotopologue ions (blue segmented traces). This reduces the signal at *m/*z 464 allocated to $C_{14}Cl_6H_{24}(NO_3^-)$ (red dashed trace) and therefore the predicted intensities of its isotopologues at higher *m/*z (teal dashed traces) are also reduced. The peaks fit at higher *m/z* are no longer greater than the observed signal.

Figure S1 demonstrates that the instrumental resolution is insufficient to fully resolve all isotopologues of each MCCP which appears in the mass spectrum. However, the signals of the most abundant MCCPs can be reliably quantified because they are much larger than nearby peaks and, due to the high number of Cl atoms in their formulas, are shifted to more negative mass defects than isobaric oxygenated organic compounds. For the four MCCP congener groups with the highest average concentrations, Table S1 shows Pearson correlation coefficients for the linear correlation across the entire campaign between the high-resolution fitted signals of the monoisotopic peak (containing zero ^{37}Cl) and its three most abundant isotopologues (containing one, two, or three ^{37}Cl atoms). For MCCP congener group quantitation the intensities of isotopologue peaks are constrained by assigning an MCCP formula to the monoisotopic peak (as shown in Fig. S1), but for this analysis the peaks were fit without assigning an MCCP formula, meaning that the signal of each isotopologue peak is allowed to vary independently of the other isotopologues.

Table S1 – Pearson correlation coefficients (R^2) of the linear relationship between the monoisotopic peak and its three most abundant isotopologues for the most concentrated MCCP congener groups

Isotopologue	$C_{14}Cl_6H_{24}$	$C_{14}Cl_7H_{23}$	$C_{15}Cl_6H_{26}$	$C_{15}Cl_7H_{25}$
$^{37}Cl_1$	0.999	0.993	0.998	0.993
$^{37}Cl_2$	0.998	0.992	0.998	0.992
$^{37}Cl_3$	0.997	0.991	0.995	0.992

Table S1 shows that the most abundant isotopologues correlate very strongly ($R^2 \geq 0.991$) with each other. This is expected for isotopologues because they are chemically identical and therefore have the same timeseries multiplied by their isotope ratios. The very strong correlations

suggest that interference from isobaric ions is minimal because any isobaric ions will have different timeseries and would decrease the strength of the correlation if they affected the quantification of MCCP isotopologue peaks they overlap with. Each MCCP formula has many isotopologues and the signals of the least abundant isotopologues are likely to be affected by interfering ions and show weaker correlations. However, the interference with less abundant isotopologues has little influence on our ability to quantify MCCP congener groups because the most abundant isotopologues account for most of the signal and can be accurately quantified. The four isotopologues shown in Table S1 account for > 80% of the total isotopologue abundance for each formula.

S2 – Pre-deployment MCCP signals

Prior to the deployment at SGP, the NO_3-CIMS measured lab air using the same inlet setup, tubing, and meshes used during the field campaign. MCCP signals were observed in lab air at levels an order of magnitude lower than the typical MCCP signals observed during the campaign. $C_{14}Cl_6H_{24}(NO_3^-)$ signals were lower than >99.9% of 5-minute average $C_{14}Cl_6H_{24}(NO_3^-)$ signals measured at SGP and multiple orders of magnitude smaller than peak $C_{14}Cl_6H_{24}(NO_3^-)$ signals. Other MCCP signals in lab air are even smaller and many MCCPs which were detected at SGP were below the detection limit in lab air. Figure S2 compares a typical 5-minute average mass spectrum recorded in lab air prior to the deployment with two 5-minute average mass spectra recorded at the SGP site. The spectra recorded at SGP contains $C_{14}Cl_6H_{24}(NO_3^-)$ signals of different magnitudes with one corresponding to the 10th percentile and the other corresponding to the 50th percentile of $C_{14}Cl_6H_{24}(NO_3^-)$ signals measured during the campaign. Even though the 10th percentile field spectrum reflects signals which are lower than the typical signals measured during the campaign, these signals are still substantially larger than the signals measured in the lab.

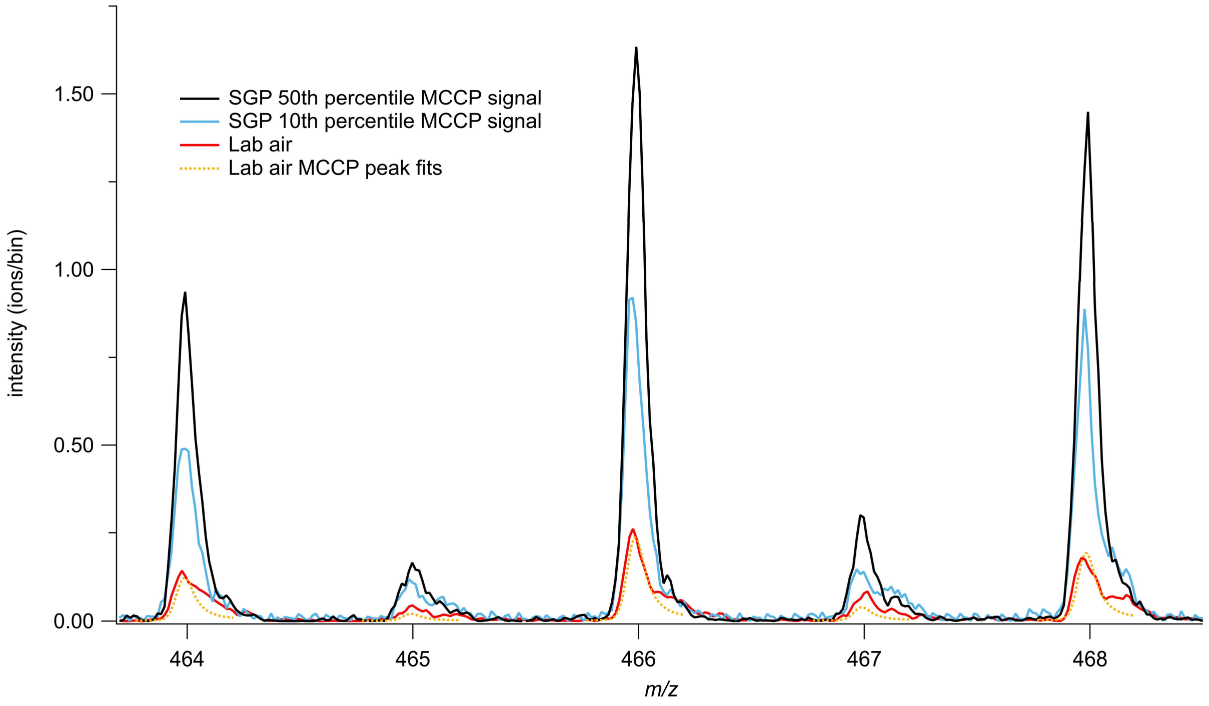

Figure S2: Overlaid five-minute average NO_3-CIMS mass spectra recorded in lab air prior to deployment (red trace) and at the SGP field site. Measurements at SGP correspond to 50th percentile (black trace) and 10th percentile (blue trace) MCCP signals. $C_{14}Cl_6H_{24}(NO_3^-)$ peak fits are shown for lab air (yellow dashed trace). This plot demonstrates that even relatively low signals at SGP are substantially larger than the signals measured in the lab.

The substantially lower signals measured in lab air allow us to rule out that the signals measured at SGP were due to instrumental contamination. This is further evidenced by the strong relationship between observed MCCPs and external meteorological variables, particularly temperature. Figure 4 shows that changes in MCCP signals closely track changes in ambient temperature. This is inconsistent with instrumental artifacts because we would expect that signals originating within the instrument would respond to changes in the temperature of the instrument

itself rather than to ambient temperature. The instrument was housed in a temperature-controlled facility and experienced minimal temperature variation.

It is not unexpected to measure a background MCCP signal. The high production volumes and historical environmental releases of MCCPs have led to widespread contamination, particularly in indoor air, leading to frequent detection of MCCPs in blank measurements.[3] While it is possible that background MCCP signals bias our measurements, this bias is minor relative to the bias introduced by the several assumptions required to estimate MCCP concentrations, e.g. that MCCPs are ionized at the collision limit and are not lost in the inlet. The signals measured in lab air correspond to $C_{14}Cl_6H_{24}$ concentrations which are on the order of 10^1 pg/m^3. If we assume that all signals measured in lab air originated in the instrument and were also present as background contamination at SGP then subtracting this "background" would have little effect on the estimated average concentration of $C_{14}Cl_6H_{24}$, which is 490 pg/m^3 (Table S2).

MCCPs are frequently detected indoors and are used in a variety of building materials.[3] It is possible that the MCCPs measured prior to deployment were not instrumental contamination and instead reflect real signals of MCCPs in the lab air. Regardless, the small bias that may be introduced by ignoring background signals is well within the uncertainty of our measurements and does not affect our conclusions, which are based on temporal trends and order-of-magnitude concentrations.

S3 – NO_3-CIMS sensitivity to MCCPs

By assuming that MCCPs are ionized at the collision limit and that they are not lost in the inlet, our calibration results in lower-limit concentration estimates. MCCP volatilities are high enough that inlet losses will be low, so the assumption of 100% transmission is not a major source of uncertainty in the overall sensitivity. It is more difficult to establish the possible range of MCCP ionization efficiencies in the NO_3-CIMS. It is likely that the average sensitivity to each congener group differs.

Ehn et al.[2] found that the NO_3-CIMS sensitivity to a fluorinated alkyl compound, perfluoroheptanoic acid, was similar to the collision-limited sensitivity to H_2SO_4. Although the MCCPs we detect are also halogenated alkyl compounds, it is possible that the presence of a carboxylic acid group in perfluoroheptanoic acid substantially increases its ionization efficiency relative to MCCPs. There is wide variation in reported sensitivities for other compounds. For example, Alage et al.[3] calculated sensitivities for small organic compounds spanning four orders of magnitude.

Using calibration assumptions that lead to lower-limit concentration estimates, the sum of all MCCPs reaches a maximum 5-minute average mass concentration on the order of 10^4 pg/m^3 during the campaign. The highest reported MCCP mass concentrations in the literature are on the order of 10^5 pg/m^3.[4] If we assume that the maximum MCCP mass concentration at SGP is at most equal to the highest concentrations reported in the literature then the potential underestimation of our calibration is at most one order of magnitude. However, we note that while it is highly unlikely that MCCP concentrations at SGP would be larger than the maximum concentrations reported in the literature, it cannot be definitively ruled out.

S4 – Congener group mass concentrations

Table S2: Average, lower quartile, median, upper quartile, and maximum mass concentrations of selected congener groups

Congener group	Average conc (pg/m^3)	Lower quartile conc (pg/m^3)	Median conc (pg/m^3)	Upper quartile conc (pg/m^3)	Maximum conc (pg/m^3)
$C_{14}Cl_6H_{24}$	490	240	360	580	5500
$C_{14}Cl_7H_{23}$	500	340	450	570	2900
$C_{14}Cl_8H_{22}$	190	150	180	230	710
$C_{15}Cl_6H_{26}$	380	210	290	450	3600
$C_{15}Cl_7H_{25}$	320	210	270	370	2200
$C_{16}Cl_6H_{28}$	190	100	140	220	1700

Table S2 shows the average, lower quartile (25th percentile), median, upper quartile (75th percentile), and maximum concentrations of the six most abundant congener groups during the entire campaign. All concentrations are calculated based on 5-minute average mass spectra. Due to the substantial uncertainties in calculated concentrations, particularly for MCCP congener groups with smaller signals, congener group concentrations are reported only for the most abundant congener groups. The reported total concentration (Sect. 3.1) includes all detected congener groups.

S5 – Timeseries of whole campaign

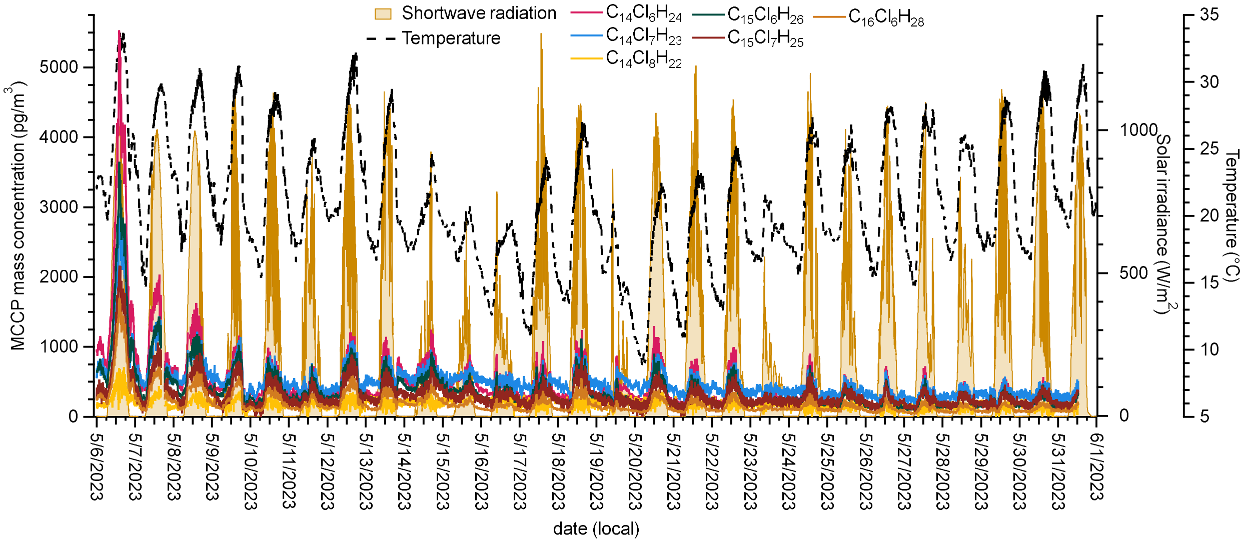

Figure S3: Timeseries of the concentration of the six most abundant MCCP congener groups, surface temperature, and shortwave solar irradiance across the entire campaign.

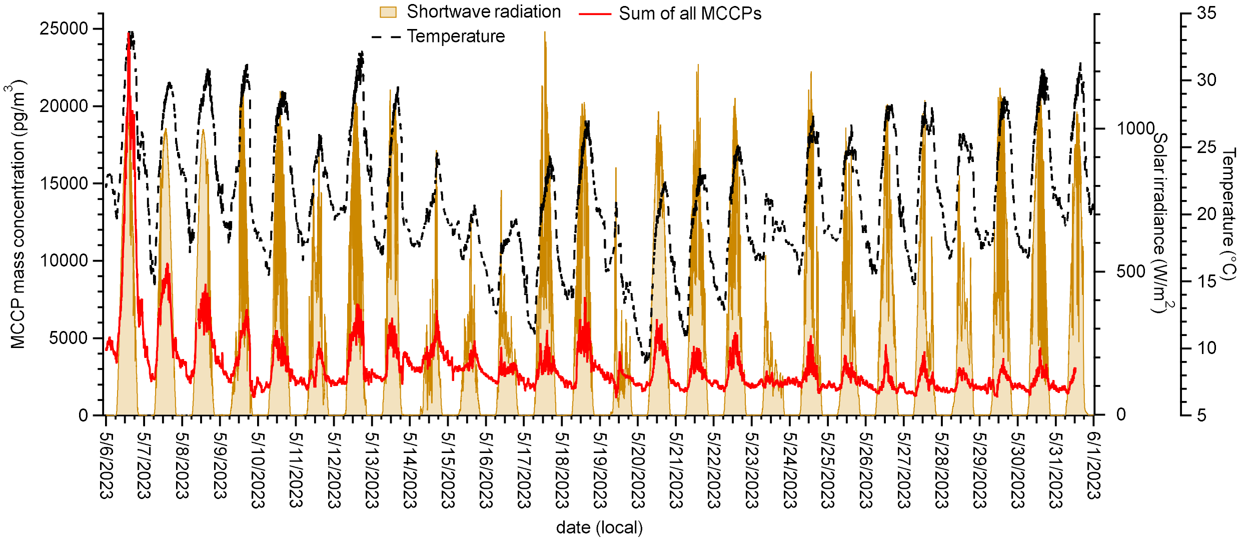

Figure S4: Timeseries of the sum concentration of all MCCPs, surface temperature, and shortwave solar irradiance across the entire campaign.

S6 – Boundary layer height diel profile

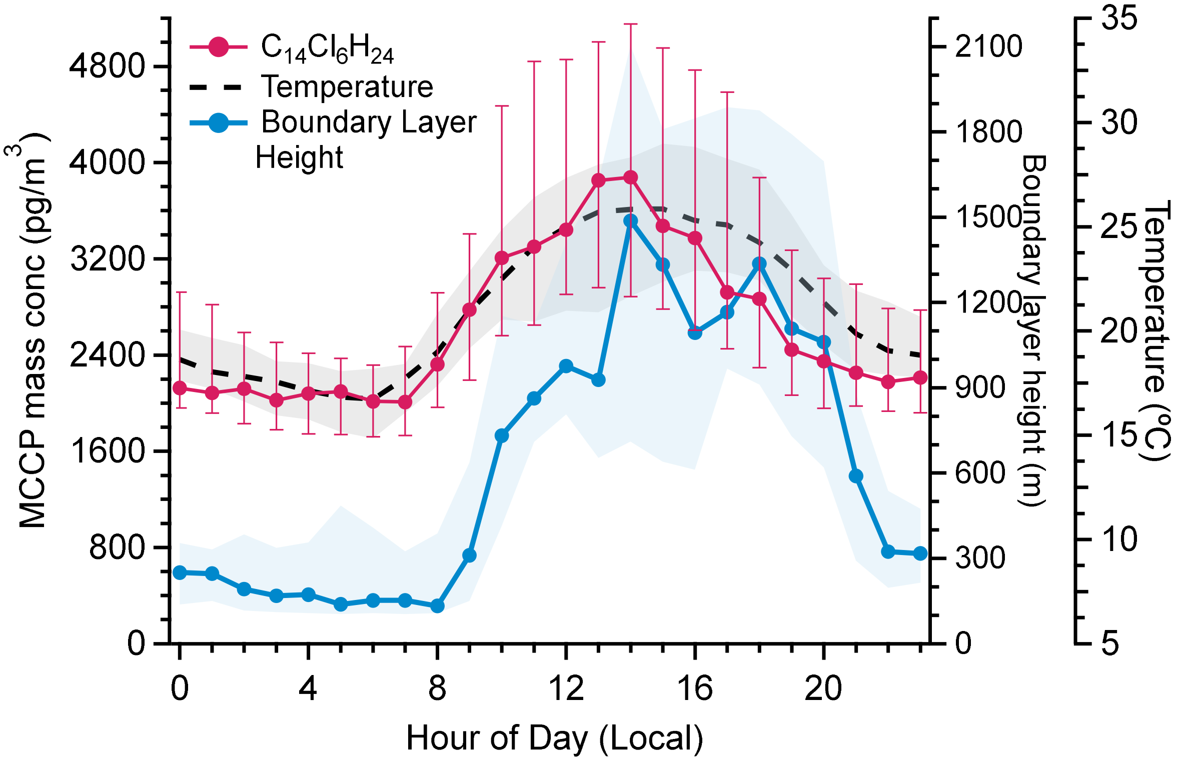

Figure S5: Median diel profiles of the sum concentration of all MCCPs, boundary layer height, and surface temperature across the entire campaign. Error bars for MCCP sum concentration and shading for boundary layer height and surface temperature represent the interquartile range.

The boundary layer height does not have a clear influence on total MCCP concentrations. This may be due to the influence of temperature. We expect higher temperatures to lead to higher boundary layer heights which could dilute MCCPs. However, higher temperatures are also expected to cause greater total gas-phase MCCP concentrations, which may offset dilution due to boundary layer dynamics.

Fig S5 shows that boundary layer heights have a consistent median diel profile which, as expected, roughly follows the median diel profile of temperature. At approximately 14:00-16:00 LT the total MCCP concentrations begin to decrease. This occurs before temperatures start to decrease significantly and coincides with the peak in boundary layer height, which could be due to dilution of MCCPs when the boundary layer height is at its maximum. Overall, temperature seems to be the more important parameter in controlling MCCP concentrations because concentrations show a much stronger relationship with temperature than boundary layer height, but due to the interrelated nature of the three variables we cannot completely separate the effects of temperature from the effects of boundary layer dynamics.

S7 – Particle fraction estimates

We calculated MCCP congener group F_p to evaluate whether the predicted trend is consistent with our measurements of gas-phase MCCP concentrations. We expect to measure higher concentrations when calculated F_p is lower and MCCPs partition more strongly into the gas phase where they can be detected by the NO_3-CIMS. This analysis assumes that changes in gas-phase MCCP concentrations are due only to partitioning between the gas and particle phases; it does not account for changes in MCCP atmospheric loading. To calculate F_p we used the Pankow model,[7] which is an equilibrium model based on saturation vapor pressure. The Harner-Bidleman model[8] has also been applied to MCCP gas-particle partitioning, and because it is also based on equilibrium partitioning theory it predicts a very similar trend in F_p, albeit with a different magnitude of F_p. For each MCCP congener group, model-derived estimates of saturation vapor pressure at 25 °C, $P°_{vap}$, and enthalpy of vaporization, ΔH_{vap},[9] were used to calculate the saturation vapor pressure, P_{vap}, across the range of temperatures observed during the campaign according the Clausius-Clapeyron equation. For each MCCP congener group i the gas-particle partitioning coefficient, $K_{p,i}$, was calculated according to Eq S2:

$$K_{p,i} = \frac{RT}{MW_i 10^6 P_{vap,i} \zeta_i} \quad (S2)$$

where is the R is the ideal gas constant (J/mol·K), T is the observed surface temperature (K), MW_i is the molecular weight (g/mol), $P_{vap,i}$ is the saturation vapor pressure at the observed temperature (Pa), and ζ_i (assumed to be unity) is the activity coefficient in the organic aerosol phase.[7,10] The values used to calculate $K_{p,i}$ are shown in Table S3. The calculated $K_{p,i}$ and observed organic aerosol mass loading, C_{OA}, at each timepoint in the campaign were then used to calculate F_p:

$$F_{p,i} = \frac{K_{p,i} C_{OA}}{1 + K_{p,i} C_{OA}} \quad (S3)$$

Table S3: Physicochemical properties used for F_p calculations

Congener group	ΔH_{vap} (kJ/mol)[a]	$logP_{vap}$, 25 °C (Pa)[a]	Molecular weight (g/mol)
$C_{14}Cl_6H_{24}$	113.3	-5.07	405.06
$C_{14}Cl_7H_{23}$	118.2	-5.66	439.50
$C_{14}Cl_8H_{22}$	122.2	-6.11	473.95
$C_{15}Cl_6H_{26}$	118.4	-5.66	419.09
$C_{15}Cl_7H_{25}$	123.5	-6.27	453.53
$C_{16}Cl_6H_{28}$	123.6	-6.25	433.11

[a]Values of physicochemical properties are from Endo.[9]

Figure S6 shows the distribution of measured concentrations corresponding to calculated F_p bins of width 0.2 for $C_{14}Cl_6H_{24}$, $C_{14}Cl_7H_{23}$, and $C_{15}Cl_6H_{26}$ congener groups. These congener groups were plotted because they are sufficiently volatile that the calculated F_p values span a range of more than 0.5. There is substantial spread in the concentrations within each bin, likely due in part to the fact that during the campaign there are real variations in MCCP atmospheric loading,

which was assumed to be constant. However, there is still a clear trend with higher concentrations of gas-phase MCCP corresponding to lower calculated F_p bins, suggesting that gas-particle partitioning is a key process controlling MCCP concentrations at SGP. Similar trends exist for the MCCP congener groups not shown in Fig. S6.

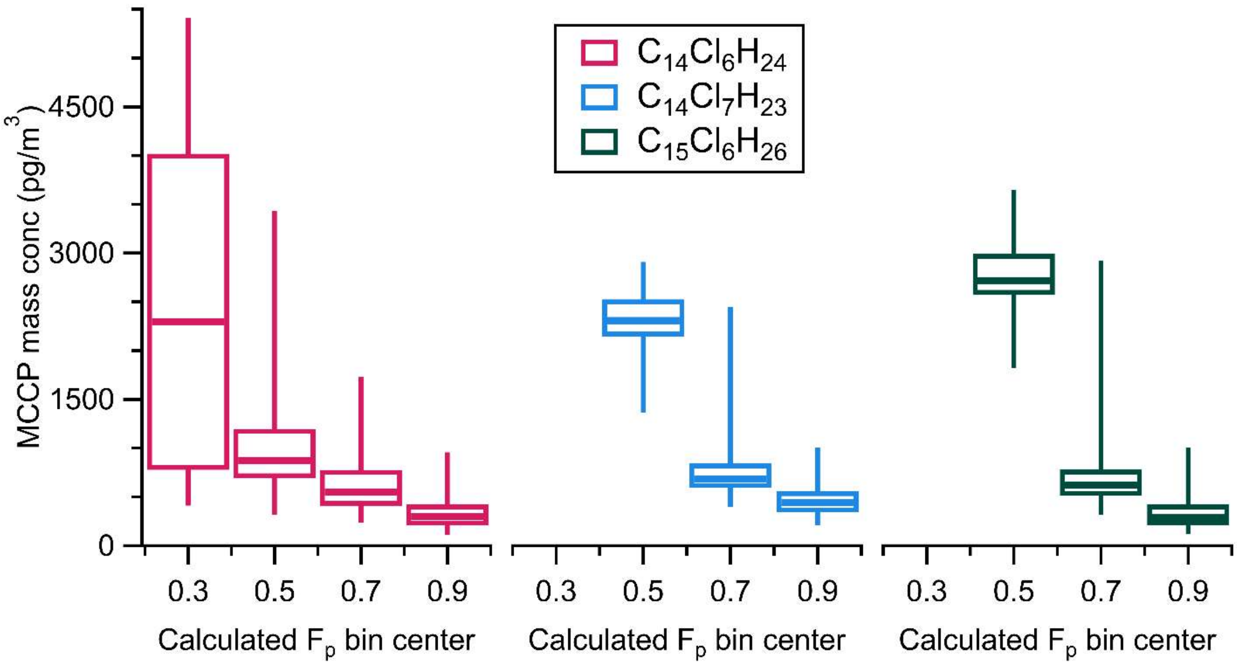

Figure S6: Box-and-whisker plots showing the distribution of $C_{14}Cl_6H_{24}$, $C_{14}Cl_7H_{23}$, and $C_{15}Cl_6H_{26}$ concentrations measured across the entire campaign grouped into calculated particle fraction bins with bin widths of 0.2. Boxes cover the interquartile range with lines showing the median. Lower and upper whiskers respectively represent the 2nd and 98th percentiles.

S8 – Timeseries of air mass change

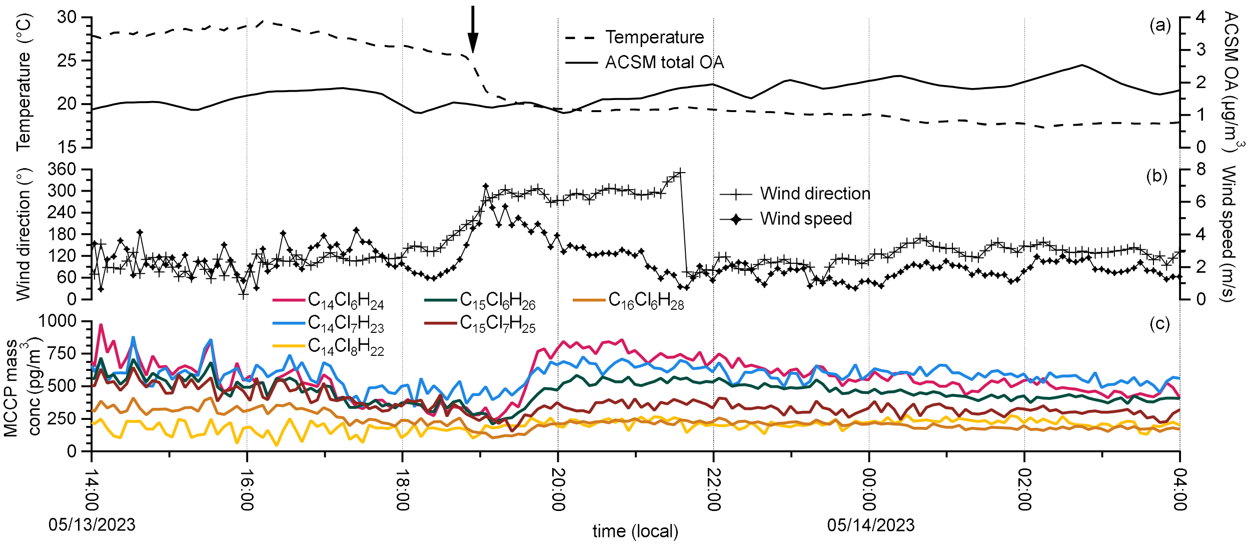

Figure S7: Timeseries from 14:00 LT May 13 to 04:00 LT May 14 of (a) surface temperature and organic aerosol mass loading, (b) wind direction and wind speed, and (c) mass concentrations of the six most abundant congener groups. The approximate time of the air mass change is indicated by the black arrow.

At approximately 18:45 LT (black arrow) there is a temperature decrease which is accompanied by a shift in wind direction. The decrease in temperature is too rapid to be explained by cooling alone and suggests that an air mass with lower temperature is arriving at the site. This is corroborated by the change in wind direction and speed. The MCCP concentrations rapidly increase as the temperature decreases. This is inconsistent with partitioning alone, which predicts that gas-phase MCCP concentrations should decrease when temperature decreases. Organic aerosol loading is low and shows a slight increase during this time. As with decreasing temperature, gas-particle partitioning alone predicts that increasing organic aerosol would decrease gas-phase MCCP concentrations, so organic aerosol also does not explain the observed behavior. The increasing MCCP concentrations during the temperature decrease suggest that the total MCCP loading has increased, likely due to a different air mass influencing the site.

S9 – Rose plots

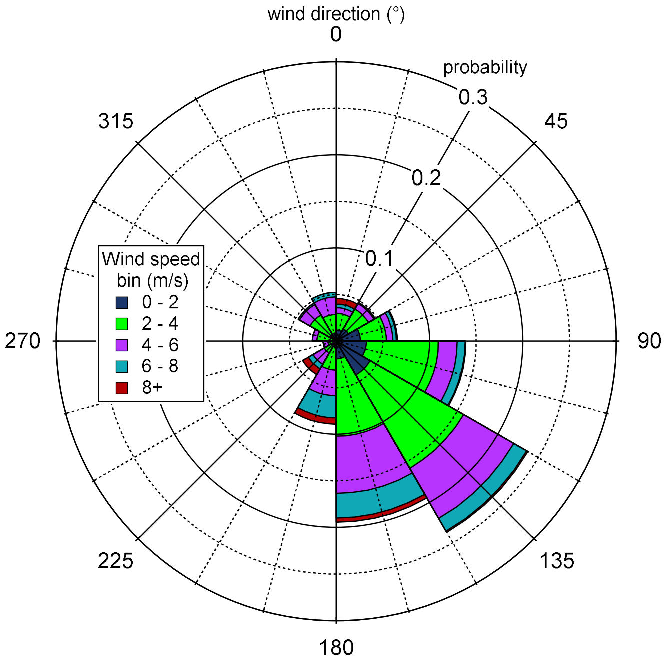

Figure S8: Wind rose during the campaign. Wind speeds are grouped into radial bins with widths of 2 m/s and wind directions are grouped into angular bins with widths of 30°. The radial axis shows the probability of observing the combination of wind speed and wind direction associated with each bin.

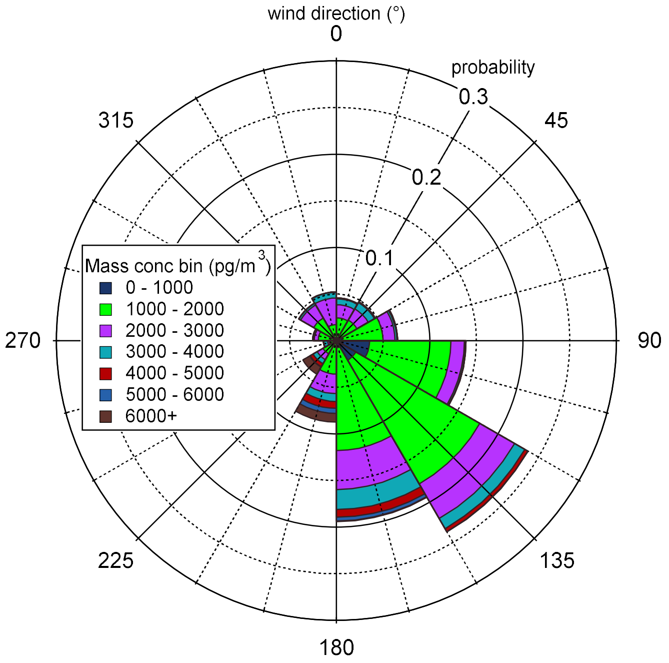

Figure S9: Pollution rose for the sum of six most abundant MCCPs during the campaign. Concentrations are grouped into radial bins with widths of 1000 pg/m^3 and wind directions are grouped into angular bins with widths of 30°. The radial axis shows the probability of observing the combination of concentration and wind direction associated with each bin.

Both the wind rose and pollution rose show that southeast wind direction have the highest probabilities of being observed. The pollution rose shows that the highest observed concentrations are associated with southwest winds, but it is difficult to tell from the rose plots that the highest observed concentrations are also associated with higher wind speeds. The nonparametric wind regression plot (main text Fig. 5) is easier to interpret than the rose plots because it shows the relationship of MCCP concentration to both wind speed and wind direction simultaneously.[11]

S10 – Proposed reaction mechanism

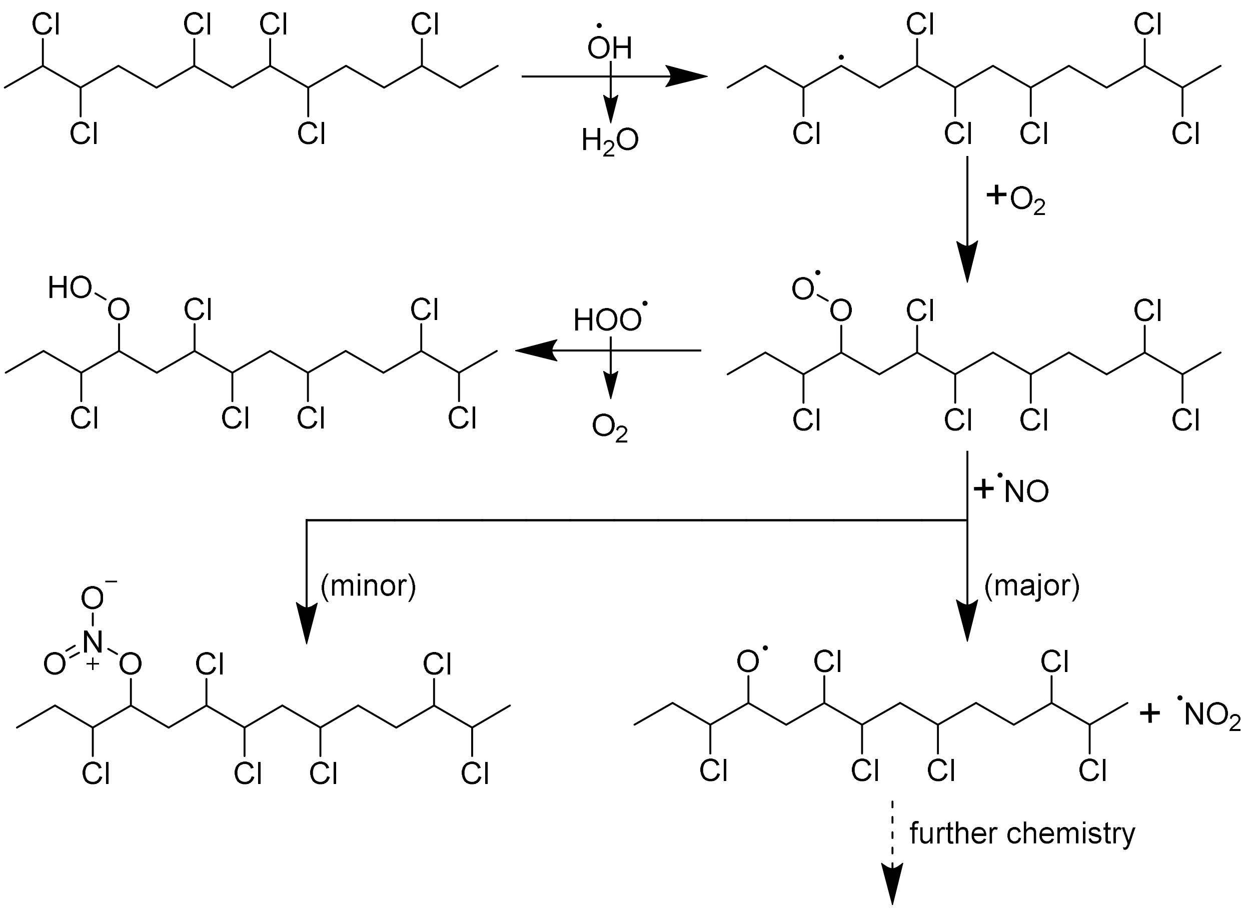

Figure S10: Proposed mechanism for the formation of $C_xCl_yH_{2x-y+2}O_2$ hydroperoxide products and $C_xCl_yH_{2x-y+1}NO_3$ organonitrate products using $C_{14}Cl_6H_{24}$ as a representative example.

In the first step, ·OH initiates oxidation of $C_{14}Cl_6H_{24}$ by abstracting an H atom, forming an alkyl radical (·$C_{14}Cl_6H_{23}$). In step two, oxygen is added to the alkyl radical, forming a peroxy radical ($C_{14}Cl_6H_{23}OO$·). The peroxy radical has several possible fates. If the peroxy radical reacts with a hydroperoxyl radical (HOO·) the hydroperoxide product ($C_{14}Cl_6H_{24}O_2$) is likely formed. If the peroxy radical reacts with ·NO the minor branch of the reaction is likely to form an organonitrate product ($C_{14}Cl_6H_{24}NO_3$). The major branch of the ·NO reaction presumably forms an alkoxy radical that will react further. The selection of the abstracted H atom in this scheme is illustrative only; each unique H atom can be abstracted by ·OH with differing rates of reaction. Similarly, the $C_{14}Cl_6H_{24}$ structure shown is only one of many possible positional isomers. The specific site of H atom abstraction and the reaction rate will depend on the locations of Cl atoms in each isomer.

S11 – Oxidized MCCP diel profiles

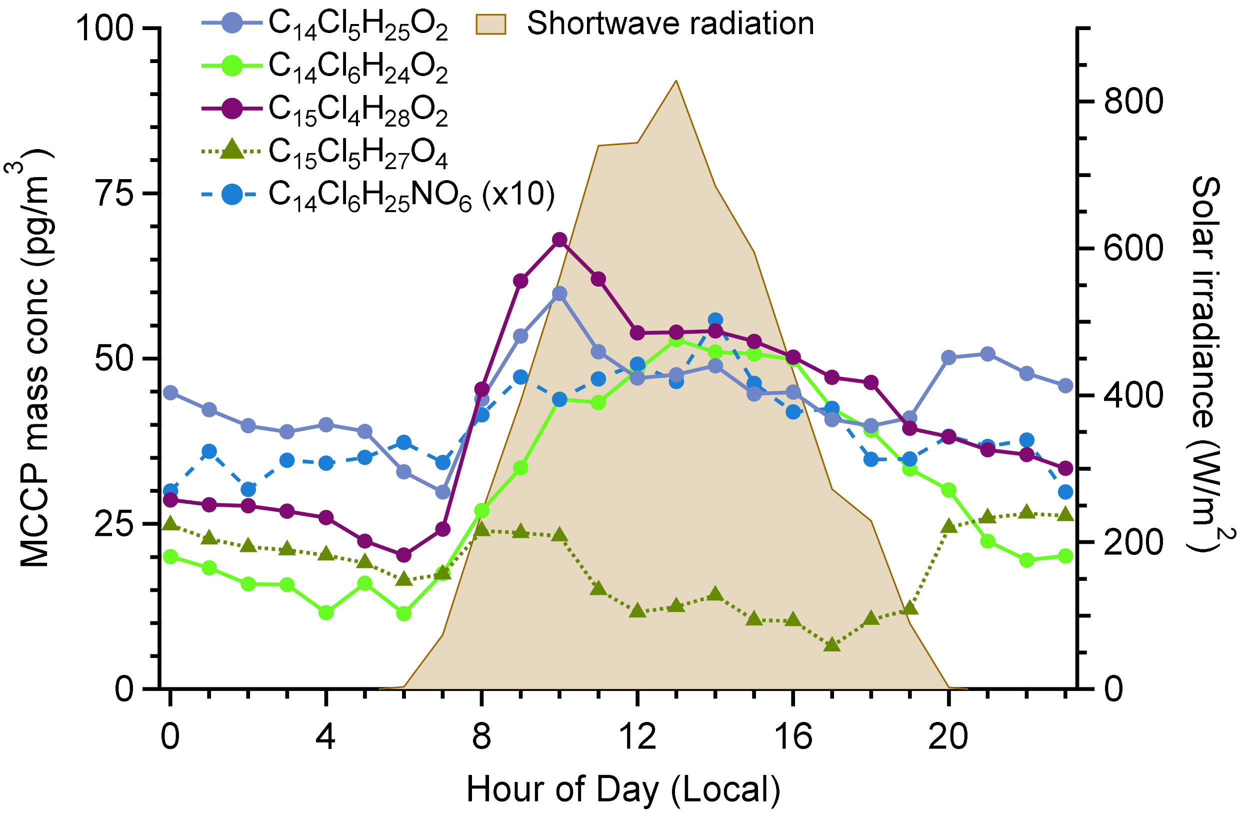

Figure S11: Diel profiles of selected oxidized MCCPs (oMCCPs). With the exception of $C_{15}Cl_5H_{27}O_4$ the diel profiles oMCCPs show a daytime increase. This coincides with the morning increase in unfunctionalized MCCPs (Fig. 2), but oMCCPs tend to peak earlier in the day than unfunctionalized MCCPs. It is likely that oMCCPs undergo similar gas-particle partitioning behavior as unfunctionalized MCCPs, but their physicochemical properties and atmospheric lifetimes are not known.

The daytime increases in oMCCPs are likely due to a combination of increased temperature, leading to oMCCPs partitioning into the gas phase, and formation of oMCCPs via daytime oxidation chemistry. While $C_{15}Cl_5H_{27}O_4$ increases slightly in the morning, it is generally lower during the day than at night. One possibility is that the rate of degradation of $C_{15}Cl_5H_{27}O_4$ by reaction with hydroxyl radicals is faster than partitioning of $C_{15}Cl_5H_{27}O_4$ from the particle phase to gas phase, leading to a net decrease in $C_{15}Cl_5H_{27}O_4$ concentration. However, understanding of MCCP atmospheric oxidation mechanisms is limited and we cannot provide a definite explanation for this observation.

References

(1) Stark, H.; Yatayelli, R. L. N.; Thompson, S. L.; Kimmel, J. R.; Cubison, M. J.; Chhabra, P. S.; Canagaratna, M. R.; Jayne, J. T.; Worsnop, D. R.; Jimenez, J. L. Methods to Extract Molecular and Bulk Chemical Information from Series of Complex Mass Spectra with Limited Mass Resolution. *Int. J. Mass Spectrom.* **2015**, *389*, 26–38. https://doi.org/10.1016/j.ijms.2015.08.011.

(2) Cubison, M. J.; Jimenez, J. L. Statistical Precision of the Intensities Retrieved from Constrained Fitting of Overlapping Peaks in High-Resolution Mass Spectra. *Atmospheric Measurement Techniques* **2015**, *8* (6), 2333–2345. https://doi.org/10.5194/amt-8-2333-2015.

(3) Yuan, B.; Muir, D.; MacLeod, M. Methods for Trace Analysis of Short-, Medium-, and Long-Chain Chlorinated Paraffins: Critical Review and Recommendations. *Anal. Chim. Acta* **2019**, *1074*, 16–32. https://doi.org/10.1016/j.aca.2019.02.051.

(4) Ehn, M.; Thornton, J. A.; Kleist, E.; Sipilä, M.; Junninen, H.; Pullinen, I.; Springer, M.; Rubach, F.; Tillmann, R.; Lee, B.; Lopez-Hilfiker, F.; Andres, S.; Acir, I. H.; Rissanen, M.; Jokinen, T.; Schobesberger, S.; Kangasluoma, J.; Kontkanen, J.; Nieminen, T.; Kurtén, T.; Nielsen, L. B.; Jørgensen, S.; Kjaergaard, H. G.; Canagaratna, M.; Maso, M. D.; Berndt, T.; Petäjä, T.; Wahner, A.; Kerminen, V. M.; Kulmala, M.; Worsnop, D. R.; Wildt, J.; Mentel, T. F. A Large Source of Low-Volatility Secondary Organic Aerosol. *Nature.* **2014**, *506* (7489), 476–479. https://doi.org/10.1038/nature13032.

(5) Alage, S.; Michoud, V.; Harb, S.; Picquet-Varrault, B.; Cirtog, M.; Kumar, A.; Rissanen, M.; Cantrell, C. A Nitrate Ion Chemical-Ionization Atmospheric-Pressure-Interface Time-of-Flight Mass Spectrometer (NO_3^- ToFCIMS) Sensitivity Study. *Atmos. Meas. Tech.* **2024**, *17* (15), 4709–4724. https://doi.org/10.5194/amt-17-4709-2024.

(6) South, L.; Saini, A.; Harner, T.; Niu, S.; Parnis, J. M.; Mastin, J. Medium- and Long-Chain Chlorinated Paraffins in Air: A Review of Levels, Physicochemical Properties, and Analytical Considerations. *Sci. Total Environ.* **2022**, *843*, 157094. https://doi.org/10.1016/j.scitotenv.2022.157094.

(7) Pankow, J. F. An Absorption Model of Gas/Particle Partitioning of Organic Compounds in the Atmosphere. *Atmos. Environ.* **1994**, *28* (2), 185–188. https://doi.org/10.1016/1352-2310(94)90093-0.

(8) Harner, T.; Bidleman, T. F. Octanol−Air Partition Coefficient for Describing Particle/Gas Partitioning of Aromatic Compounds in Urban Air. *Environ. Sci. Technol.* **1998**, *32* (10), 1494–1502. https://doi.org/10.1021/es970890r.

(9) Endo, S. Refinement and Extension of COSMO-RS-Trained Fragment Contribution Models for Predicting the Partition Properties of C10–20 Chlorinated Paraffin Congeners. *Environ. Sci. Process. Impacts.* **2021**, *23* (6), 831–843. https://doi.org/10.1039/D1EM00123J.

(10) Donahue, N. M.; Robinson, A. L.; Stanier, C. O.; Pandis, S. N. Coupled Partitioning, Dilution, and Chemical Aging of Semivolatile Organics. *Environ. Sci. Technol.* **2006**, *40* (8), 2635–2643. https://doi.org/10.1021/es052297c.

(11) Henry, R.; Norris, G. A.; Vedantham, R.; Turner, J. R. Source Region Identification Using Kernel Smoothing. *Environ. Sci. Technol.* **2009**, *43* (11), 4090–4097. https://doi.org/10.1021/es8011723.
